# Altered lipid homeostasis is associated with cerebellar neurodegeneration in SNX14 deficiency

**DOI:** 10.1172/jci.insight.168594

**Published:** 2024-04-16

**Authors:** Yijing Zhou, Vanessa B. Sanchez, Peining Xu, Thomas Roule, Marco Flores-Mendez, Brianna Ciesielski, Donna Yoo, Hiab Teshome, Teresa Jimenez, Shibo Liu, Mike Henne, Tim O’Brien, Ye He, Clementina Mesaros, Naiara Akizu

**Affiliations:** 1 Perelman Center for Cellular and Molecular Therapeutics, Children’s Hospital of Philadelphia, Philadelphia, Pennsylvania, USA.; 2 Department of Pathology and Laboratory Medicine and; 3 Department of Systems Pharmacology and Translational Therapeutics, Perelman School of Medicine, University of Pennsylvania, Philadelphia, Pennsylvania, USA.; 4 Institute for Translational Medicine and Therapeutics, University of Pennsylvania, Philadelphia, Pennsylvania, USA.; 5 The Graduate Center of the City University of New York, Advanced Science Research Center, New York, New York, USA.; 6 Department of Cell Biology, UT Southwestern Medical Center, Dallas, Texas, USA.; 7 Ph.D. Program in Biology, The Graduate Center of the City University of New York, New York, New York, USA.

**Keywords:** Neuroscience, Lysosomes, Monogenic diseases, Neurodegeneration

## Abstract

Dysregulated lipid homeostasis is emerging as a potential cause of neurodegenerative disorders. However, evidence of errors in lipid homeostasis as a pathogenic mechanism of neurodegeneration remains limited. Here, we show that cerebellar neurodegeneration caused by Sorting Nexin 14 (SNX14) deficiency is associated with lipid homeostasis defects. Recent studies indicate that SNX14 is an interorganelle lipid transfer protein that regulates lipid transport, lipid droplet (LD) biogenesis, and fatty acid desaturation, suggesting that human SNX14 deficiency belongs to an expanding class of cerebellar neurodegenerative disorders caused by altered cellular lipid homeostasis. To test this hypothesis, we generated a mouse model that recapitulates human SNX14 deficiency at a genetic and phenotypic level. We demonstrate that cerebellar Purkinje cells (PCs) are selectively vulnerable to SNX14 deficiency while forebrain regions preserve their neuronal content. Ultrastructure and lipidomic studies reveal widespread lipid storage and metabolism defects in SNX14-deficient mice. However, predegenerating SNX14-deficient cerebella show a unique accumulation of acylcarnitines and depletion of triglycerides. Furthermore, defects in LD content and telolysosome enlargement in predegenerating PCs suggest lipotoxicity as a pathogenic mechanism of SNX14 deficiency. Our work shows a selective cerebellar vulnerability to altered lipid homeostasis and provides a mouse model for future therapeutic studies.

## Abstract 2

SNX14-deficient cerebella show lipid storage and clearance defects in predegeneration, providing evidence for lipotoxicity-induced neurodegeneration and a model for therapeutic studies.

## Graphical abstract

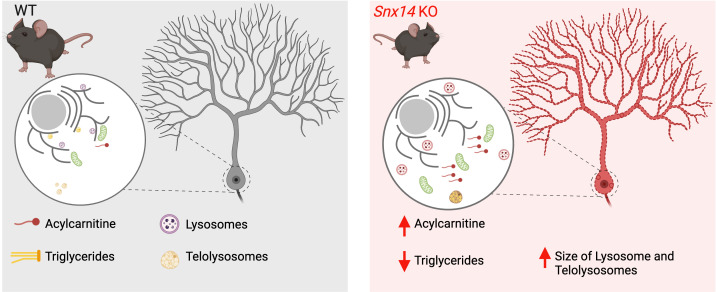

## Introduction

Neurodegenerative disorders are characterized by a progressive loss of specific neuronal types often associated with the accumulation of toxic protein aggregates (1). To better understand disease mechanisms and find therapeutic alternatives, the field has principally focused on the study of protein quality control pathways, including autophagy (2, 3). In contrast, little attention has been paid to lipid homeostasis pathways despite their well-established association with neurodegeneration and relevance for the function and integrity of cellular organelles (4–7).

Genetic disorders affecting regulators of lipid homeostasis often show neurodegeneration, particularly affecting the cerebellum and spinal cord (8, 9). The cerebellum integrates motor function with cognition, emotion, and language, and its dysfunction is documented in a wide spectrum of neurological disorders (10–12). Among cerebellar disorders, childhood-onset spinocerebellar ataxias are the most severe. In addition to impaired motor coordination and balance, spinocerebellar ataxia in children is often accompanied by additional neurologic and systemic symptoms, including neurodevelopmental delay and intellectual disability (13, 14). Recent efforts that combine patient registry assemblies with advances in sequencing technologies are revealing a new class of childhood cerebellar neurodegenerative disorders caused by disfunction of lipid homeostasis pathways (8, 15).

Mutations in *Sorting Nexin 14* (*SNX14*) are the cause of a childhood-onset ataxia known as Spinocerebellar Ataxia Recessive 20 (SCAR20), characterized by progressive cerebellar degeneration and severe intellectual disability (16–18). We previously discovered that SCAR20 is associated with enlarged lysosomes and altered autophagy in neural cells derived from patients (16). These findings were also reproduced in patient skin fibroblasts and SNX14-deficient U2OS cell lines but deemed secondary to defects in cholesterol distribution and neutral lipid metabolism (19). Subsequent studies identified SNX14 as a regulator of cholesterol homeostasis in 2 independent genome-wide perturbation screens (20, 21). Although the mechanisms by which SNX14 regulates cholesterol trafficking are still unknown, recent reports demonstrate that SNX14 is recruited to the endoplasmic reticulum-lipid droplet (ER-LD) contact sites to facilitate the incorporation of fatty acids (FA) into triglycerides (TGs) of growing LDs (22). In this process, SNX14 interacts with SCD1, an ER-anchored FA desaturase, to cooperate in FA incorporation into LDs (23). Consequently, SNX14-deficient cells show enhanced toxicity to saturated FAs and defective FA-stimulated LD biogenesis (22, 23). Furthermore, recent structural predictions suggest that SNX14 and its SNX-RGS family members may be involved in intracellular lipid transfer (24). However, it is currently unknown if the role of SNX14 in lipid homeostasis regulation is implicated in the pathogenesis of SCAR20.

To shed light on the cellular and molecular mechanisms that lead to cerebellar degeneration and intellectual disability in SNX14 deficiency, we generated the first *Snx14* full-body KO mouse (*Snx14*-KO) that survives to adulthood. Our work shows that *Snx14*-KO mice recapitulate cerebellar atrophy as well as motor and cognitive defects of patients with SCAR20. Whereas cerebellar atrophy is associated with Purkinje cell (PC) degeneration, forebrain regions responsible for cognitive behavior remain protected from neurodegeneration. Guided by transcriptomic analyses that pointed to lipid dysregulation as a potential cause of selective cerebellar degeneration, we identify tissue-specific alterations of lipid profiles in *Snx14*-KO mice. Particularly, nondegenerating *Snx14*-KO cerebral cortices exhibit reduced phosphatidylethanolamine (PE) levels that may be associated with synaptic dysfunction, while accumulation of acylcarnitines (AcCa-s) is unique to predegenerating cerebella and likely associated with selective cerebellar neurodegeneration. Finally, we show that SNX14 deficiency reduces LD content and causes lipid storage defects in cerebellar PCs. Together, our work provides evidence for the involvement of lipid homeostasis defects in selective neurodegeneration and uncovers lipid targets for therapeutic interventions.

## Results

### SNX14 deficiency causes partial embryonic lethality and developmental delay in mice.

**Figure 1:**
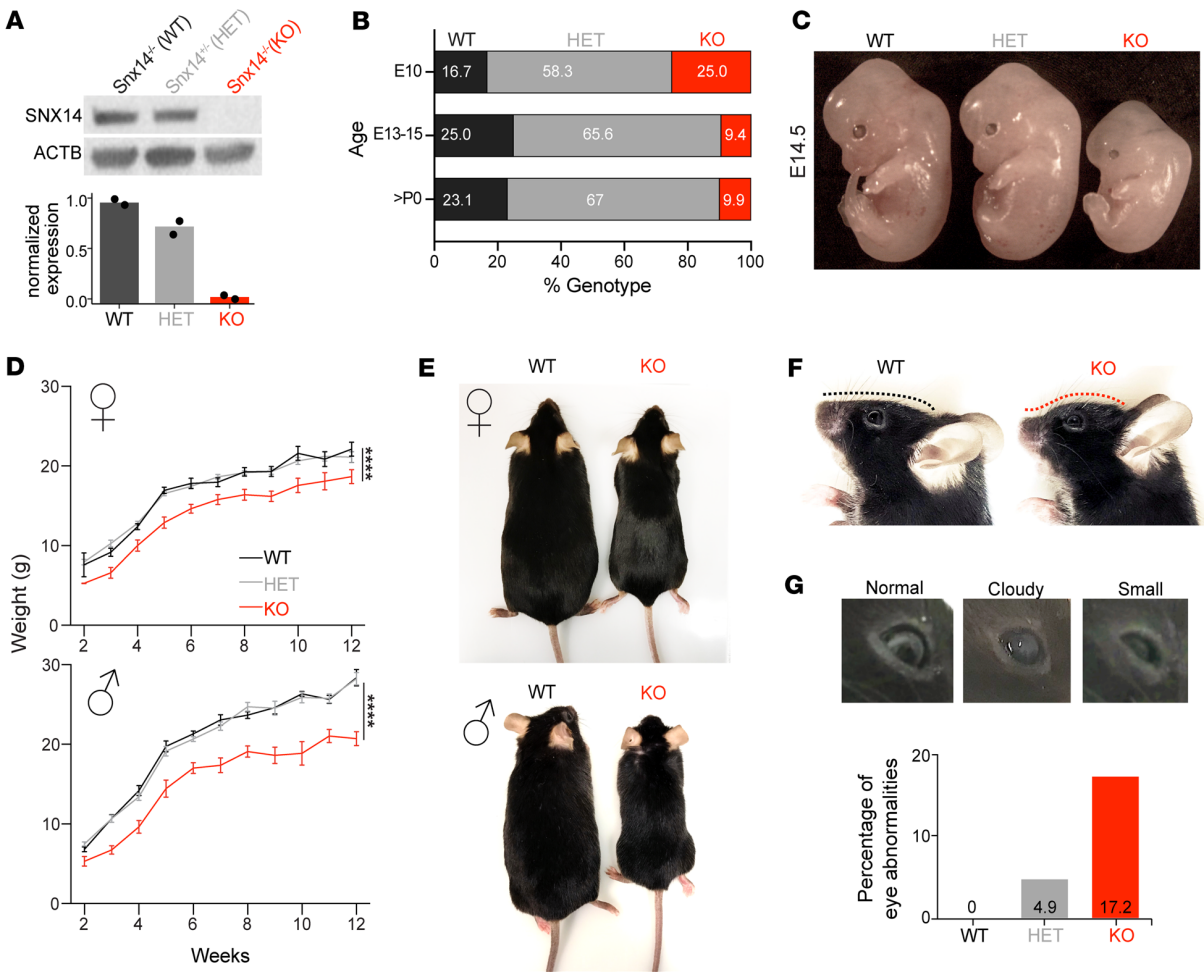
SNX14-deficient mice show developmental delay and atypical facial features. (**A**) Representative Western blot (WB) images show loss of SNX14 expression in *Snx14*-KO mice tissue. β-Actin (ACTB) was used as loading control. Bar graph shows WB band densitometry quantification of SNX14 relative to ACTB. *n* = 2 for each genotype. (**B**) Percentage of embryos/mice with the indicated genotypes obtained from heterozygous parent mattings. The χ^2^ test shows siginficant discrepancy between >P0 observed and expected values (*P* = 0.001) indicating embryonic lethality of KOs. E10, *n* = 12; E13–15, *n* = 32; >P0, *n* = 91. (**C**) Representative image of WT, HET, and KO E14.5 embryos showing smaller size of KOs. (**D**) Growth curves show consistently lower body weight of 2- to 12-week-old *Snx14*-KO males and females. Data represent mean ± SEM of *n* ≥ 3. Two-way ANOVA shows siginficant effect of genotype. ****P < 0.0001. (**E**) Representative images of 9-month-old WT and KO littermates of each sex. (**F**) Representative images showing the atypical face with forehead protrusion of 6-month-old KO mice (red line) compared with WT littermate. (**G**) Representative images of 8-month-old KO mice showing eye abnormalities, including cataracts (cloudy) and microphthalmia (small). Bar graph shows percentages of mice with eye abnormality for each genotype.

SCAR20 patients share clinical features of developmental delay and perinatal onset neurodegeneration of the cerebellum. Previous work suggests that the severity of developmental phenotypes is species specific, with SNX14-deficient mice showing fully penetrant embryonic lethality, while dogs and zebrafish display neurological and metabolic defects reminiscent of SCAR20 patients (25). However, by randomly introducing a frameshift 1 bp deletion in the exon 14 of *Snx14* (c.1432delG; p.Glu478Argfs*18), we successfully generated SNX14-deficient mice (*Snx14*-KO) that are viable and thrive despite a complete loss of SNX14 protein and 90% reduction of the transcript when the mutation is in homozygosity (Figure 1A and Supplemental Figure 1, A–D; supplemental material available online with this article; https://doi.org/10.1172/jci.insight.168594DS1).

Although *Snx14*-KO mice survive to adulthood, we noticed that they were born in lower than the expected Mendelian ratio (observed 9.9% versus expected 25%) (Figure 1B). To test if the reduced birth ratio was due to embryonic lethality, we genotyped embryos produced by heterozygous breeding pairs and uncovered that about half of *Snx14*-KO embryos die between E10 and E15. The other half were distinguishable by their small size, a feature that persisted throughout neonate and adulthood (Figure 1, C–E). Notably, similar to SCAR20 patients (16–18), adult *Snx14*-KO mice showed dysmorphic facial features characterized by an upturned nose, bulging forehead, and eye defects (Figure 1, F and G). These data indicate that SNX14 deficiency in mice causes developmental delay phenotypes reminiscent of SCAR20.

### Snx14-KO mice display motor and cognitive behavioral defects.

**Figure 2:**
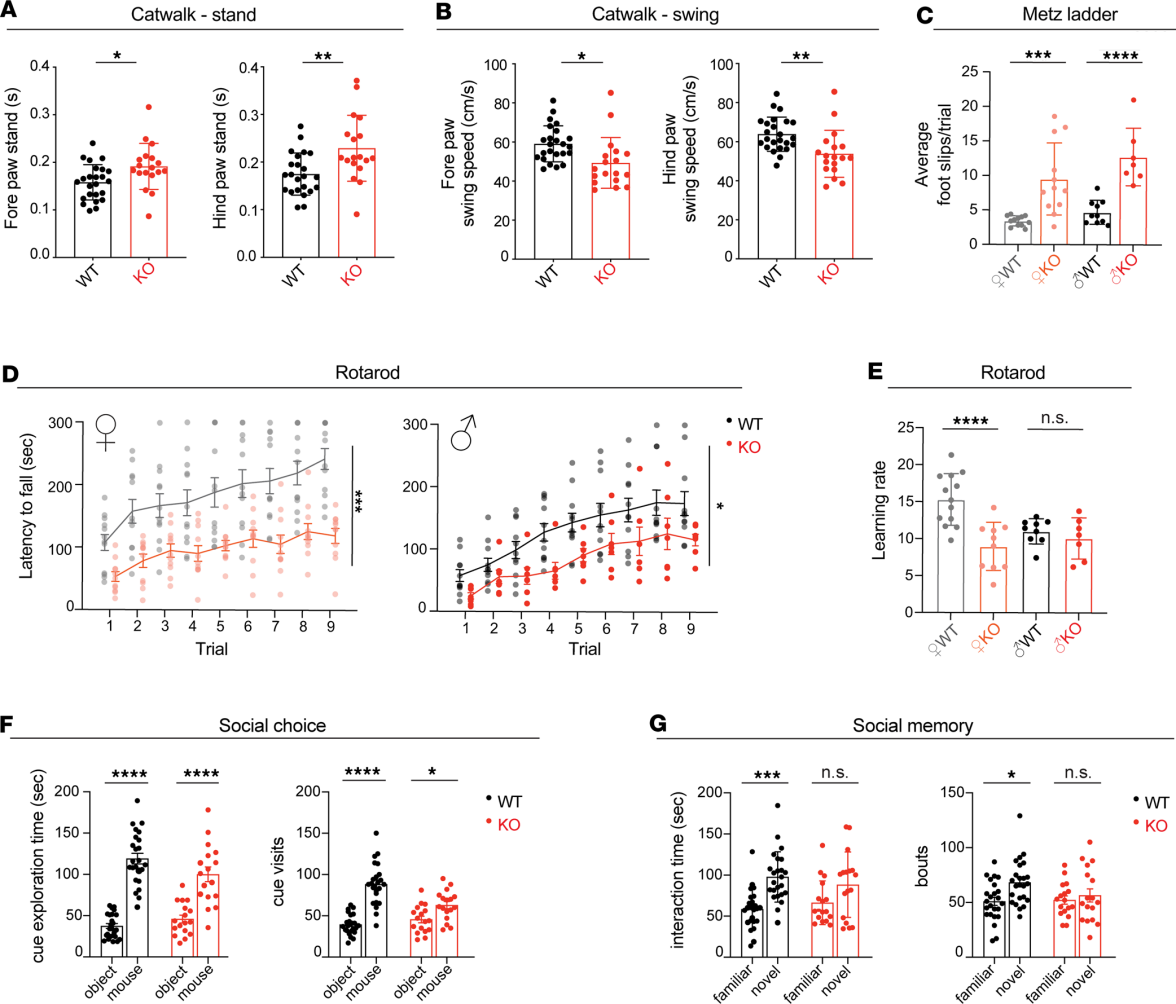
SNX14 deficiency in mice recapitulates motor and behavioral deficits of SCAR20. (**A** and **B**) Catwalk analysis shows altered gait of KO mice with a longer stand (**A**) and shorter swing (**B**) than WT mice. Data are shown as mean ± SEM of *n* = 24 WT and *n* = 18 KO mice. Two-tailed Welch’s *t* test. (**C**) Metz ladder rung test shows altered limb placing and coordination of KO males and females. Data are shown as mean foot slip of 5 trials performed in consecutive days ± SEM of *n* = 10 WT males, *n* = 7 KO males, *n* = 12 WT females, and *n* = 12 KO females. Two-way ANOVA followed by Šidák’s test. (**D**) Accelerating rotarod reveals defects in motor performance of KO mice in the 9 trials performed over 3 consecutive days. Data are shown as mean latency to fall ± SEM of *n* = 11 WT males, *n* = 7 KO males, *n* = 13 WT females, and *n* = 11 KO. Two-way ANOVA shows siginficant effect of genotype. (**E**) KO females show impaired learning rate on accelerating rotarod performance over time (between trial 1 and 9). Data are shown as mean learning rate ± SEM of *n* = 9 WT males, *n* = 7 KO males, *n* = 13 WT females, and *n* = 10 KO females. Two-way ANOVA followed by Šidák’s test. (**F** and **G**) Three-chamber social interaction test showing similar preference for a mouse over an object between WT and KO mice (**F**) but impaired social novelty preference in KO mice (**G**). Data are shown as mean ± SEM of *n* = 24 WT and *n* = 17 KO. Two-way ANOVA followed by Tukey’s test. *P < 0.05, **P < 0.01, ***P < 0.001, ****P < 0.0001.

Unlike SCAR20 patients who show severe gait abnormalities typical of cerebellar degeneration, *Snx14*-KO mice were undistinguishable from their WT littermates based on their home cage walking activity. However, catwalk gait analysis revealed a mild gait disruption characterized by longer paw stand time and faster swing speed of the limbs (Figure 2, A and B). Functional gait disruption was seen on the horizontal Metz ladder, where mice cross a series of rungs separated by varying distances. Here, *Snx14*-KO mice had siginficantly more foot slips than control mice (Figure 2C). Moreover, *Snx14*-KO mice underperformed when challenged with complex motor tasks that require coordination and balance. On the accelerating rotarod, *Snx14*-KO mice showed difficulty maintaining balance (Figure 2D) similar to other cerebellar ataxia mouse models (26). In addition, the accelerating rotarod procedure was performed in 3 consecutive days to assess motor learning. While WT mice improved their performance over trial, *Snx14*-KO learning rate was low, especially for females (Figure 2E).

Given that intellectual disability is also a hallmark of SCAR20, we wondered whether *Snx14-*KO mice had broader behavioral deficits. To answer this question, we performed a test for social preference and recall (27). During a choice phase of the procedure, *Snx14*-KO mice showed typical preference for a social cue relative to an inanimate object (Figure 2F). However, in the recall phase, *Snx14*-KO mice failed to discriminate between a familiar and a novel mouse (Figure 2G). Thus, *Snx14*-KO mice showed similar preference to the social cue, but their lack of preference toward exploration of the novel mouse suggests a social memory deficit likely caused by dysfunction of brain regions, including the cerebellum (28, 29).

### Behavioral defects are associated with cerebellar atrophy.

**Figure 3:**
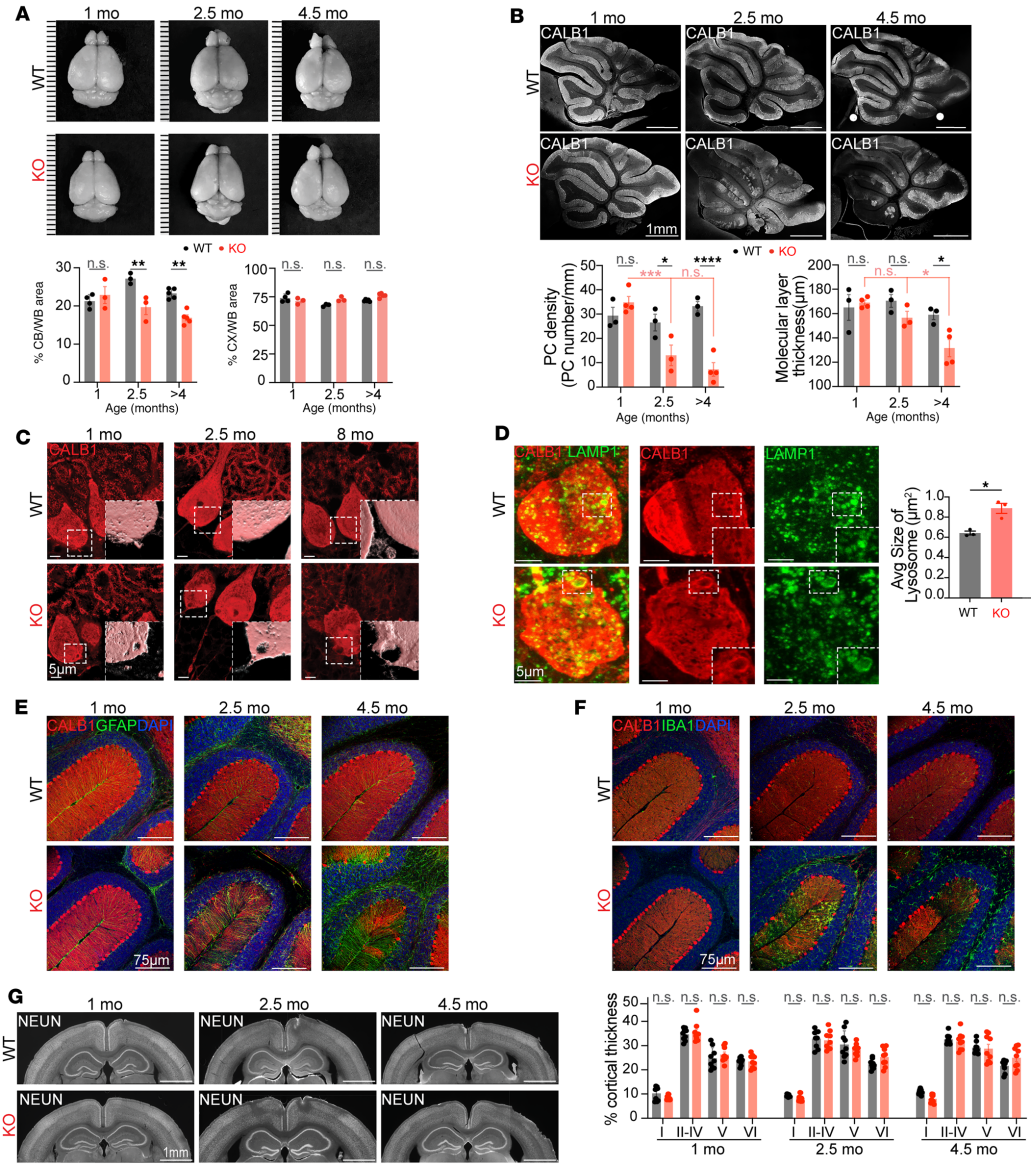
SNX14 deficiency causes selective cerebellar degeneration. (**A**) Representative brain images from WT and KO mice at indicated age shows shrinkage of KO cerebellum (CB) over time. Ruler marks separated by 1 mm. Bar graphs show percentage area of CB or cerebral cortex (CX) relative to the whole brain (WB) in *n* = 3–5 mice. Two-way ANOVA followed by Šidák’s test. (**B**) Representative cerebellar sagittal sections immunostained with PC-specific anti-CALB1 antibody reveal progressive loss of PCs in KO mice. Bar graphs show PC linear density (right) and thickness of the molecular layer (left) in the cerebellar lobule III of *n* = 3–4 mice. Two-way ANOVA followed by Šidák’s test. (**C**) Representative immunostaining of PCs with anti-CALB1 antibody reveals progressive accumulation of vacuoles in KO mice. (**D**) Immunostaining of PCs with anti-CALB1 and lysosomes with anti-LAMP1 show enlarged lysosomes in KO mice. Bar graph shows average lysosome size per mouse. *n* = 3 mice (in WT, 29 PCs and 4,033 lysosomes were counted; in KO, 30 PCs and 3,247 lysosomes were counted). Two-tailed *t* test. (**E** and **F**) Representative immunostaining showing progressive accumulation of astrocytes labeled with anti-GFAP (**E**) and microglia with anti-IBA1 (**F**) in degenerating KO cerebella (base of lobules III and IV). (**G**) Coronal sections of cerebral cortices immunostained with anti-NeuN do not show differences between WT and KO mice. Bar graphs show percentage thickness occupied by each cortical layers (I–VI) in 4–5 cortical regions of 2 mice per genotype and age. Two-way ANOVA followed by Šidák’s test. In all graphs, data represent mean ± SEM. *P < 0.05, **P < 0.01, ****P < 0.0001. Scale bars: 1 mm, 5 μm, 75 μm.

Having established that SNX14-deficient mice recapitulate developmental, motor, and behavioral delays of SCAR20, we looked for the underlying neuropathologic causes. Similar to humans, in mice, SNX14 is widely expressed in the developing and adult brain, with a slight enrichment in older brains (Supplemental Figure 2A). In line with the expression pattern, the gross brain morphology of *Snx14*-KO mice appeared normal during the first month of life but showed defects as mice became older. Specifically, we found that *Snx14*-KO mice had smaller cerebella than WT littermates starting at 2.5 months of age while forebrain areas were mostly intact (Figure 3A), suggesting that the cerebellum is particularly vulnerable to SNX14 deficiency.

### SNX14 deficiency causes selective PCs degeneration.

To further determine vulnerabilities of SNX14 deficiency at a cellular level, we histologically analyzed cerebellar and forebrain tissue. Recent single-cell transcriptomics data show that, within the cerebellum, *Snx14* expression is enriched in Golgi cells and PCs (30) (Supplemental Figure 2B). Accordingly, RNAscope in situ hybridization showed an enrichment of *Snx14* in PCs (Supplemental Figure 2, C and D). PCs are some of the largest neurons in the nervous system, and their loss is a hallmark of cerebellar ataxias (31). Thus, we first analyzed PCs in 1-, 2.5-, and 4-month-old cerebellar sections by immunofluorescence (IF) staining with Calbindin 1 (CALB1) antibody. At 1 month of age, both WT and *Snx14*-KO cerebellar stainings showed perfectly aligned somas in the PC layer and PC dendrites extended into the molecular layer (ML). However, by 2.5 months of age, patches of missing PCs were evident in *Snx14*-KO cerebella (Figure 3B). Quantification of PC number per mm of PC layer confirmed siginficantly lower PC density in lobule III of 2.5- and 4-month-old *Snx14*-KO cerebella compared with WT (Figure 3B, bottom left graph). The loss of PCs in *Snx14*-KO cerebella was followed by a reduced thickness of the ML that was first detectable at 4 months of age (Figure 3B, bottom right graph). Upon closer examination of CALB1 staining, we identified vacuole-like structures within *Snx14*-KO PC dendrites and soma (Figure 3C and Supplemental Figure 3A). Although these vacuoles were more abundant and larger in older cerebella, they were sparsely detected in 1-month-old PCs, suggesting that these vacuoles may be a pathological sign that precedes PC neurodegeneration. Remarkably, IF staining with anti-LAMP1 antibody revealed that enlarged vacuoles overlap with lysosomal structures and *Snx14*-KO PCs display larger lysosomes in comparison with WT (Figure 3D).

Given that PC degeneration is often followed by disorganization of Bergmann glia (BG) processes and gliosis, we also immunostained sagittal cerebellar sections with anti-GFAP and anti-IBA1 antibodies. Concurrent with PC loss, anterior lobes of 2.5-month-old *Snx14*-KO cerebella showed abnormal branching of GFAP^+^ BG processes (Figure 3E) and an accumulation of IBA1^+^ microglia within the ML (Figure 3F). Moreover, we found that reactive astrocytes progressively accumulate near the PC layer from 2.5 to 4.5 months of age (Figure 3E). Interestingly, these findings were specific of the anterior lobes of *Snx14*-KO cerebella while posterior lobes (VIII and IX) did not show signs of neurodegeneration until 11 months of age (Supplemental Figure 3, B–D). Notably, we did not detect neuronal loss or signs of gliosis in cortical and hippocampal regions of the forebrain (Figure 3G and Supplemental Figure 4, A–C).

Taken together, our results indicate that, despite the wide expression of SNX14 in the whole brain, the forebrain and posterior cerebellum are protected from neurodegeneration, while anterior PCs selectively neurodegenerate in SNX14-deficient mice after 2 months of age.

### Lipid response genes are dysregulated in predegenerating Snx14-KO mice cerebella.

**Figure 4:**
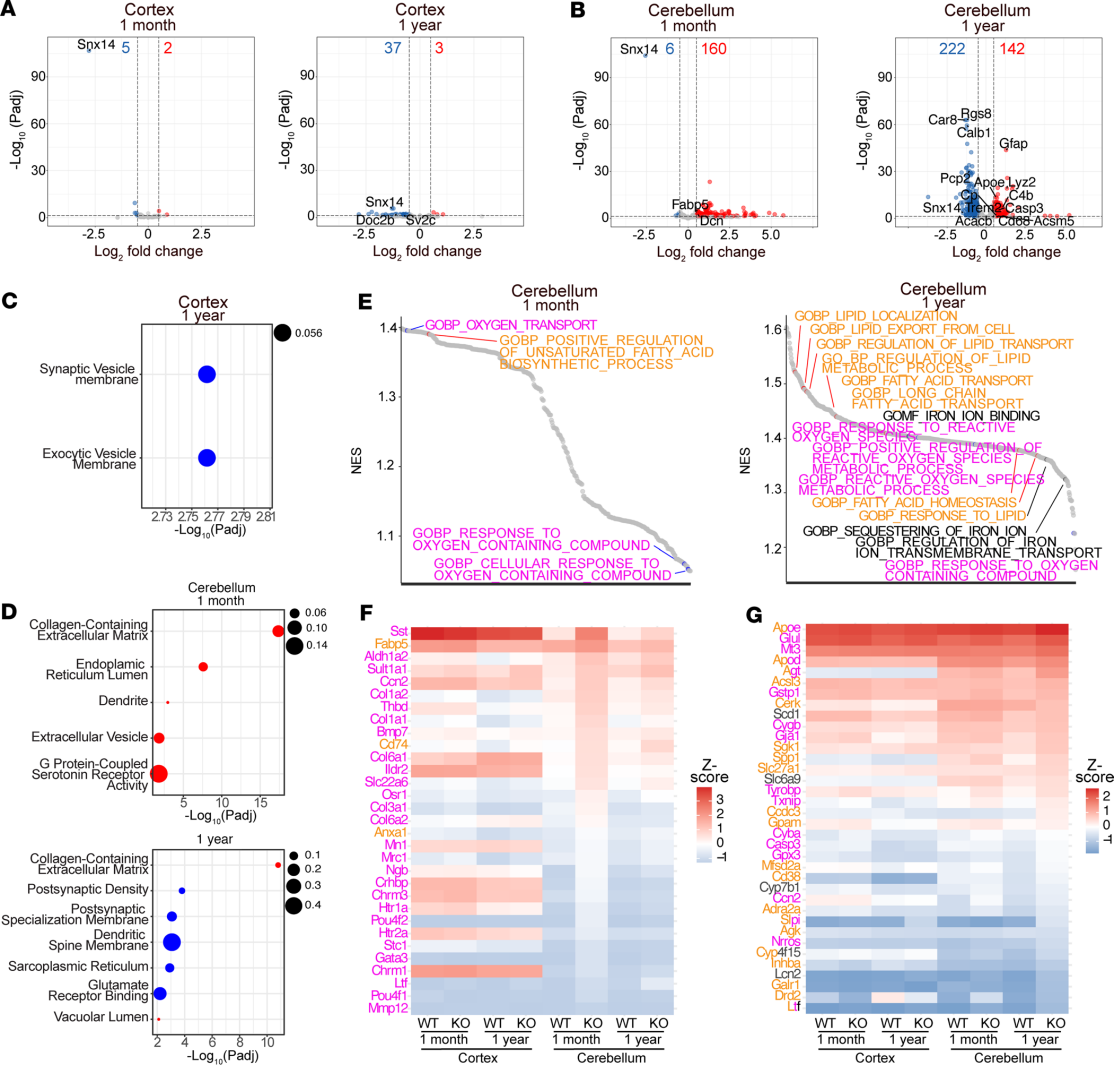
Genes involved in lipid response are differentially expressed in SNX14-deficient cerebella. (**A** and **B**) Volcano plots of differentially expressed genes (DEGs) in the cortex and cerebellum of WT versus KO mice at 1 month and 1 year. Dashed lines indicate statistical siginficance cut off (–log_10_[*P*_adj_] > 1.301 and log_2_[FC] = ± 0.5). Number of siginficantly down- and upregulated genes are displayed on the top of each plot in blue and red, respectively. (**C** and **D**) Dot plots of gene ontology (GO) enrichment analysis of the DEGs, with selected down- and upregulated GO categories marked in blue and red, respectively. Dot size indicates proportion of DEGs relative to the total number of genes in each category. (**E**) Waterfall plots of Gene Set Enrichment Analysis (GSEA) of cerebellum-specific siginficant gene ontology terms. Terms in orange, magenta, and black are related to lipid, oxygen, and iron, respectively. (**F**) Heatmap of the top 20 leading edge genes of each term displayed in the 1-month cerebellum GSEA shown in **E**. (**G**) Heatmap of the top 10 leading edge genes of each term displayed in the 1-year Cerebellum GSEA shown in **E**.

To gain insights into the molecular mechanisms of selective cerebellar PC degeneration, we next analyzed the transcriptome of *Snx14*-KO mice cerebella at pre- and postdegenerating stages (1-month-old and 1-year-old, respectively) and compared them with cerebral cortices, which do not show signs of neurodegeneration. After RNA-Seq, we defined differentially expressed genes (DEG) as those showing absolute log_2_(FC) > 0.50 with adjusted *P* value (*P*_adj_) < 0.05 between *Snx14-*KO and WT tissue. As expected, *Snx14* was downregulated in all *Snx14-*KO samples (Figure 4, A and B, and Supplemental Figure 1, C and D). Few differences between *Snx14-*KO and WT cerebral cortex transcriptomes were detected at < 1 month of age (7 DEGs including *Snx14*) and only 37 downregulated and 3 upregulated DEGs at 1 year of age (Figure 4A). None of these DEGs suggest changes in specific cell type composition, which is consistent with the lack of neurodegeneration or neuroinflammation in our histological analyses (Supplemental Data 1). We then tested if cortical DEGs were enriched in specific cellular and molecular functional annotations. Given the short list of DEGs at 1-month-old cortices, we only performed functional annotation analysis on the 37 downregulated genes at 1 year of age. Results reveal a siginficant enrichment for genes involved in synaptic vesicle membrane (i.e., *Doc2b, Sv2c*) (Figure 4, A and C). Accordingly, SNX14 has been shown to promote synaptic transmission in mouse cortical neuronal cultures (32). To further validate these data, we analyzed cortical sections by IF staining of pre- and postsynaptic puncta markers and by Western blot (WB) analysis of synaptic vesicle protein, SV2A, levels. Remarkably, IF and WB results were consistent with a reduction of excitatory and inhibitory synaptic puncta (Supplemental Figure 4D) and SV2A protein levels (Supplemental Figure 4E), suggesting that cognitive behavioral deficits in SNX14 deficiency are likely caused by defects in synaptic signaling of forebrain cortical neurons.

**Figure 5:**
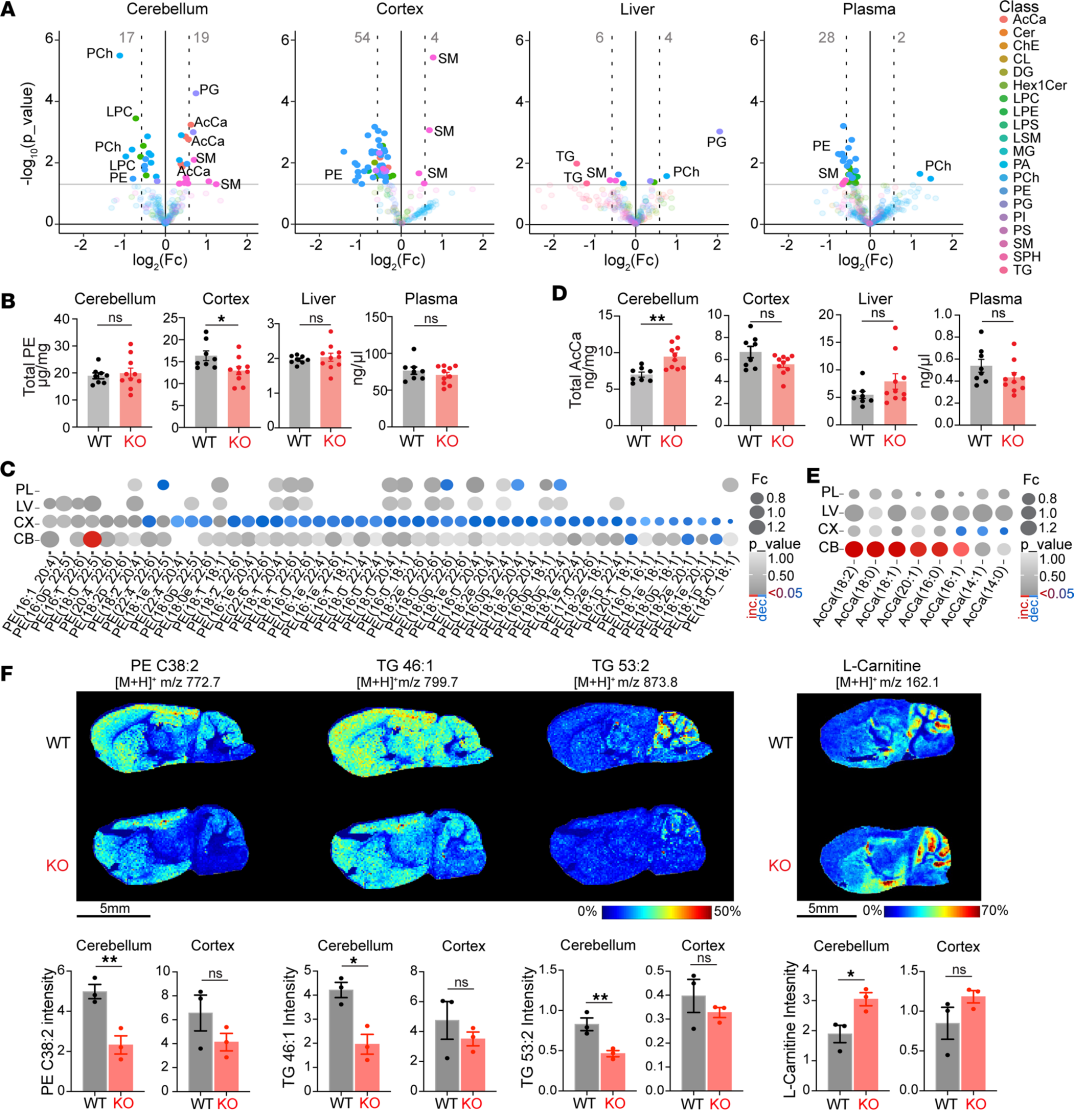
Unique deregulation of lipid metabolites in predegenerating KO cerebella. (**A**) Volcano plots show deregulated lipids in 2-month-old *Snx14*-KO cerebellum (CB), cerebral cortex (CX), liver, and plasma. Horizontal gray lines indicate *P* < 0.05 cut-off. Data show increased concentrations of acylcarnitine (AcCa) species specifically in KO CB. (**B**) Bar graphs show total PE concentrations per tissue in *n* = 8 WT and *n* = 10 KO mice. Two-tailed *t* test. PEs are siginficantly reduced in *Snx14-*KO CX. (**C**) Dot plot depicting fold change (FC) (proportional to dot size) and *P* value (in gray intensity scale) of PE species detected in cerebral cortices for all analyzed tissues. Red dots represent siginficantly increased lipids, while blue dots represent siginficantly decreased lipids. (**D**) Bar graphs show total AcCa concentrations in *n* = 8 WT and *n* = 10 KO mice. Two-tailed *t* test. AcCa-s are siginficantly increased only in KO CB. (**E**) Dot plot depicting FC and *P* value of AcCa species detected in cerebellar samples for all analyzed tissues. Red dots represent siginficantly increased lipids, while blue dots represent siginficantly decreased lipids. (**F**) MALDI-MS imaging of brain cryosections show reduction of PE C38:2, TG 46:1, and TG 53:2 and cerebellar accumulation of L-carnitine in KO. The molecules were revealed in positive ion mode using DHB matrix, and the *m*/*z* (mass/charge ratio) of [M + H]^+^ are indicated. Heatmap colors depict the relative abundance of each metabolic species. Bar graphs show cerebellar or cortical intensity of each lipid species in *n* = 3 per genotype. In all panels, data are shown as mean ± SEM. *P < 0.05, **P < 0.01. Key of each lipid class can be found Supplemental Figure 6 legend. Scale bar: 5 mm.

Unlike cerebral cortices, cerebellar transcriptomes were markedly different between *Snx14*-KO and WT mice, with 160 upregulated and 6 downregulated DEGs at 1 month of age and 142 up- and 222 downregulated DEGs at 1 year of age in *Snx14*-KO mice (Figure 4, B and D–G and Supplemental Figure 5A). We reasoned that the increase in the amount of downregulated DEGs from 1 month to 1 year of age could reflect the progressive PC loss in *Snx14*-KO cerebella. Accordingly, most downregulated DEGs in 1-year *Snx14*-KO cerebella correspond to PC markers, such as *Calb1*, *Pcp2*, *Car8*, and *Rgs8* (Figure 4B). To unbiasedly test this observation, we analyzed 1-year-old DEGs for functional annotation enrichments. Furthermore, we compared DEGs with a list of a recently reported mouse cerebellar single nuclear RNA-Seq data set (30). Results confirm that downregulated DEGs were enriched in genes predominantly expressed in PCs (Supplemental Figure 5, B and C). In contrast, most of the upregulated DEGs genes were sparsely expressed across various cerebellar cell types, with a group of them typically expressed in astrocytes and macrophage/microglia (*Lyz2*, *C4b*, *Cd68*, *Trem2*, *ApoE*, *Gfap*) or associated with cell death (Casp3) (Figure 4B and Supplemental Figure 5, B and C). Notably, 1-month-old DEGs were not enriched for PC or astroglia-specific functional annotations, indicating a later onset of neurodegeneration, consistent with our histological analyses (Figure 4B, Figure 5D, Supplemental Figure 5C). Additionally, functional annotation analysis revealed enrichments of genes localized in synaptic, dendritic, and ER compartments in pre- and postdegenerating cerebella (Figure 4D and Supplemental Data 2).

Given the lack of neurodegenerative signs in histology or transcriptomics data (Figures 3 and 4), we anticipated that DEGs at 1-month-old cerebella could point us to the molecular causes that precede PC neurodegeneration. However, considering that PCs only constitute ~1% of the total cerebellum, transcriptomic changes in PCs may only contribute to small fold changes in bulk transcriptomics data. To account for these small changes, we analyzed our RNA-Seq data with Gene Set Enrichment Analysis (GSEA). Interestingly, GSEA revealed cerebellar-specific enrichments in biological processes involved in oxidative stress (e.g., “response to oxygen containing compounds” in 1-month-old cerebella and “response to reactive oxygen species” in 1-year-old cerebella), FA or lipid homeostasis regulation (e.g., “response to positive regulation of unsaturated FA biosynthetic process” in 1-month-old cerebella and “response to lipid” in 1-year-old cerebella), and iron accumulation (e.g., “regulation of iron ion transmembrane transport” and “iron ion binding” in 1-year-old cerebella) (Figure 4, E–G). Remarkably, genes contributing to enrichment of lipid and iron terms in predegenerating cerebella include upregulated *Fabp5,* which encodes a protein involved in interorganelle lipid transport (33), and *Dcn* encoding a protein released by cells dying by ferroptosis (34) (Figure 4, B and E–G). These data suggest that lipid homeostatic defects may precede selective cerebellar degeneration in SNX14 deficiency.

### Snx14 deletion alters lipid metabolite levels in a tissue-specific manner.

We next set out to analyze lipid metabolite composition of predegenerating cerebella in 2-month-old WT and *Snx14*-KO mice by unbiased lipidomic analysis. As a control of a nondegenerating tissue, we included their cerebral cortices in the analysis. Since the liver is a lipid-rich organ with high content of TGs stored in LDs, we included liver lipid extracts as a control for lipid metabolite detection. Finally, to distinguish tissue-specific lipids from those circulated by their blood supply, we also extracted plasma lipids from circulating blood.

The lipid extracts were analyzed by ultraperformance liquid chromatography–high-resolution mass spectrometry (UPLC-HRMS) as previously described (35), and after normalization with lipid internal standards, we quantitatively identified > 200 lipid species per sample (Supplemental Data 3). Overall, *Snx14*-KO and WT tissues had similar total lipid concentrations (Supplemental Figure 6A), and each tissue analyzed was distinguishable by their relative lipid class abundance. For instance, the liver displayed the highest abundance of TGs while the cerebral cortex and cerebellum had phosphatidylcholines (PCh) as the most abundant lipid class (Supplemental Figure 6B). These data are consistent with the literature (36, 37), thus validating our methodology.

Next, we aimed to determine how SNX14 deficiency affects tissue-specific lipid composition. To this end, we compared the concentration of each lipid specie in *Snx14*-KO and corresponding WT tissue. Given SNX14’s role in facilitating the incorporation of FAs into TG during LD biogenesis (22), we hypothesized that SNX14 deficiency would result in a depletion of TG levels. Although TGs were undetectable in all cerebellar and cortical samples, *Snx14*-KO livers displayed a siginficant reduction of TGs (Figure 5A and Supplemental Figure 6B), further confirming our hypothesis and the reliability of our lipidomic analysis.

Additionally, results show that the cerebral cortex and cerebellum are the tissues with the largest amount of altered lipid species upon SNX14 depletion (Figure 5A). Using *P* < 0.05 as a cutoff, we identified 58 and 36 altered lipid species in cerebral cortices and cerebella, respectively. Furthermore, only cerebellar samples clustered by genotype in a principal component analysis (Supplemental Figure 6C), suggesting SNX14 has a larger effect on lipid homeostasis in the cerebella than in the other tissues we analyzed.

Among the 58 altered lipids in cerebral cortices, 54 had lower concentrations in KOs, and 40 belong to the PE class (Figure 5, A–C). PEs provide fluidity and curvatures to membranes, and this may facilitate vesicular budding and membrane fusion essential for synaptic vesicle formation (38). Thus, changes in PE species may alter cerebral cortex–dependent behaviors and executive functions in SNX14 deficiency. The remaining 4 lipid species had higher concentrations in *Snx14*-KO than in WT and all were sphingomyelins (SMs) (Figure 5A). Similarly, *Snx14*-KO cerebella exhibited increased levels of total SM concentrations (Figure 5A and Supplemental Figure 6D). While some PEs were lower in *Snx14*-KO cerebella, these did not influence total PE concentration (Figure 5, A and B). In addition, *Snx14*-KO cerebella were distinguishable from the cortex, liver, and plasma, given the increased levels of several acylcarnitine (AcCa) species (Figure 5, A, D, and E). Specifically, 6 of 16 increased lipids in *Snx14*-KO cerebella were AcCa-s. This accounted for the majority of AcCa-s detected in cerebella (6 out of 8) and resulted in an overall increase of total AcCa concentration in *Snx14*-KO cerebella. *Snx14*-KO cerebellar samples were also the ones with the largest amount of accumulated lipid species among all the analyzed tissues.

To further determine region-specific differences in lipid metabolite abundance in situ, we next analyzed brain sections with matrix-assisted laser desorption ionization and MS imaging (MALDI-MSI). Results uncover differences between WT and *Snx14*-KO brain lipid patterns consistent with UPLC-HRMS results, including reduced PE C38:2 levels (Figure 5F). Furthermore, 2 TGs were reduced in *Snx14*-KO cerebella, 1 of which (TG 53:2) is specific to the outermost layer of the cerebellar cortex comprised by PC soma and dendrites (Figure 5F). L-carnitine signal also overlapped this area and was more intense in *Snx14*-KO than WT (Figure 5F). Given L-carnitine’s involvement in AcCa metabolism, this increase in signal may be associated with the accumulation of AcCa in this cerebellar region.

Taken together, the bulk and in situ lipidomic analyses show tissue-specific lipid metabolite defects, including a cerebellar-specific AcCa accumulation that may be associated with the selective cerebellar neurodegeneration characteristic of SNX14 deficiency.

### Snx14 deletion impairs lipid storage in vivo.

**Figure 6:**
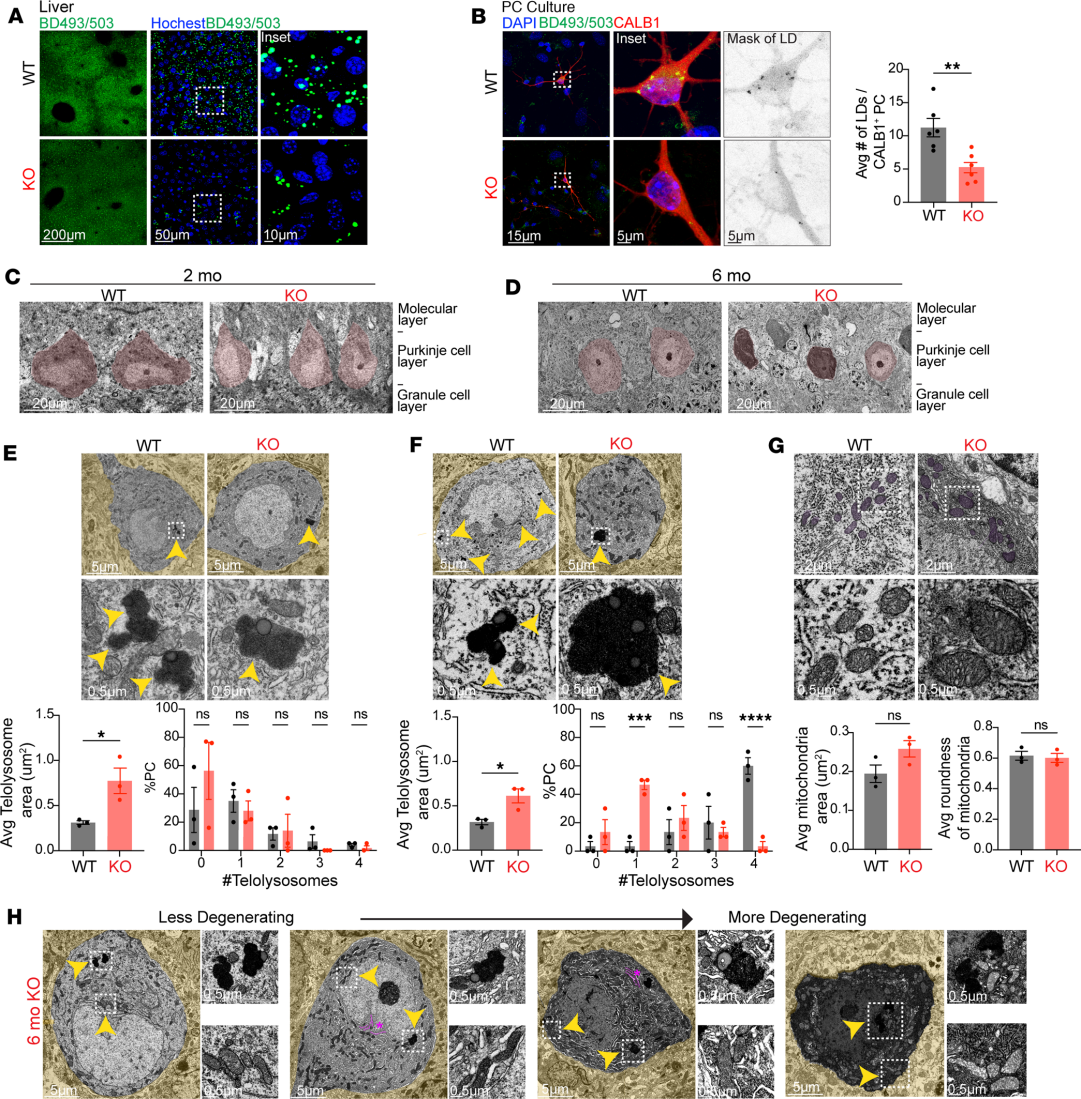
Lipid storage organelles are affected in SNX14-deficient tissue. (**A**) Representative BODIPY 493/503 (BD493) labeling shows less lipid droplets (LDs) in 2-month-old KO mice liver sections. (**B**) Representative BD493 and anti-CALB1 labeling shows less LDs in *Snx14-*KO primary cerebellar culture PCs. Bar graphs show average number of LDs per CALB1^+^ PC in *n* = 6 mice per genotype used for PC cultures. Total number of CALB1^+^ PC quantified: *n* = 69 WT and *n* = 50 KO. Two-tailed *t* test. (**C** and **D**) Representative TEM image of PC layer in WT and KO mice at 2 (**C**) and 6 (**D**) months of age. (**E**) Representative TEM images of PCs show less but larger telolysosomes in 2-month-old KO mice. Bottom graphs show the average area of telolysosomes (left) and the percentage of PCs with indicated number of telolysosomes (right) in *n* = 3 mice per genotype (6–10 PCs per mouse). Two-tailed *t* test (left) and 2-way ANOVA followed by Šidák’s test (right). (**F**) Representative TEM image of PCs showing less but larger telolysosomes in 6-month-old KO mice. Bottom graphs show the average area of telolysosomes (left) and percentage of PCs with indicated number of telolysosomes (right) in *n* = 3 mice per genotype (6–10 PCs per mouse). Two-tailed *t* test (left) and 2-way ANOVA followed by Šidák’s test (right). (**G**) Representative TEM image of PC mitochondria at 6 months of age. Bottom bar graphs show the average area (left) and roundness (right) of mitochondria in *n* = 3 mice per genotype (10 PCs per mouse). Two-tailed *t* tests. (**H**) Representative TEM images show a spectrum of less to more degenerating PCs from 6-month-old KO mice. Yellow arrowheads point to insets of enlarged telolysosomes in **E** and **F**, and mitochondria and enlarged telolysosomes in **H**. ER swelling highlighted in magenta and indicated with an asterisk. In all panels, data represent mean ± SEM. *P < 0.05, **P < 0.01, ***P < 0.001, ****P < 0.0001. Scale bars: 200 μm, 50 μm, 15 μm, 5 μm, 20 μm, 5 μm, 2 μm, 0.5 μm.

Under conditions of high energy demand or nutrient deprivation, AcCa-s carry FAs into the mitochondria for β oxidation. Elevated concentrations of AcCa, however, can become cytotoxic and disrupt mitochondrial function. Here, LDs are vital for storing excessive FAs and preventing AcCa-induced toxicity (39). Accordingly, increased AcCa and decreased TG levels in *Snx14*-KO cerebella could be a consequence of defects in LD biogenesis. In line with this idea, SNX14 interacts with LDs, and its deficiency leads to impaired LD content and morphology in cell cultures (22). Thus, we investigated whether *Snx14* deletion alters LD biogenesis in the cerebellum by staining *Snx14*-KO and WT mice cerebella with Bodipy 493/503 (BD493), a fluorescent dye that stains neutral lipids typically stored in LDs. As a control, we stained the liver, a LD-rich tissue, and detected abundant BD493^+^ LDs in WT sections. Results also reveal a prominent reduction of LD amounts in *Snx14*-KO liver (Figure 6A), which is consistent with the reduction of TG levels in *Snx14*-KO liver lipidomics (Figure 5A and Supplemental Figure 6B). These data suggest that SNX14 is necessary for LD biogenesis in vivo — at least in the liver.

Next, we focused our attention on the cerebellum. Here, BD493 staining showed few, if any, structures resembling LDs, even in WT PCs (Supplemental Figure 7A). To further explore the possibility that *Snx14* deletion affects LD biogenesis in PCs, we stimulated LD biogenesis in cerebellar cultures by supplementation with oleic acid (OA). As expected, OA induced LD biogenesis in WT PCs (Figure 6B). In contrast, the number of LDs detected in *Snx14*-KO PCs was half the number in WT PCs (Figure 6B), indicating that SNX14 is necessary for LD biogenesis in PCs as well.

To assess for LDs or alternative lipid storage defects in *Snx14*-KO PCs in tissue, we analyzed cerebellar sections by transmission electron microscopy (TEM) after imidazole-buffered osmium tetroxide staining to highlight LDs (40) (Figure 6, C–H). An overview of PC integrity in TEM images confirmed that most *Snx14*-KO PCs are still intact at predegenerating ages (2 months) (Figure 6C) while a gradient of degenerating PCs is observed at 6 months of age (Figure 6, D and H). Again, TEM studies failed to identify LDs in the cerebellum at predegenerating (2 months) or postdegenerating stages (6 months). Nonetheless, TEM results reveal that, at predegenerating ages, *Snx14*-KO PCs have less and larger telolysosomes, which are lipid-rich lysosomal storage organelles (Figure 6, E and F, and Supplemental Figure 7B). Interestingly, this is consistent with larger lysosome compartments we observed in *Snx14*-KO PCs (Figure 3D) and in SCAR20 patient neural cell lines (16). These results suggest that SNX14 may have a specialized function regulating lipid clearance or storage through the lysosomal compartment in PCs.

Fewer yet enlarged telolysosomes in PCs and elevated AcCa-s at predegenerating *Snx14*-KO cerebella suggest that lipid homeostasis defects underlay PC degeneration in SNX14 deficiency (39). Nevertheless, increased AcCa can also be a consequence of mitochondrial damage. Therefore, to determine if AcCa accumulation is a consequence of lipid storage defects or caused by mitochondrial damage, we assessed mitochondrial ultrastructure of PCs by TEM. Results show mostly intact mitochondria in predegenerating *Snx14*-KO PCs (Figure 6G and Supplemental Figure 7C), suggesting that AcCa accumulation is the result of lipid storage and clearance defects, not mitochondrial damage. Furthermore, at 6 months, ultrastructure analysis also revealed a progressive enlargement of ER as PCs degenerate. Damaged mitochondria with enlarged and disorganized cristae were only observed in most degenerated PCs (Figure 6H).

Together, our work indicates that lipid storage and clearance defects are associated with PC neurodegeneration in SNX14 deficiency, contributing to the expanding list of neurodegenerative disorders associated with lipid homeostasis defects.

## Discussion

SNX14 deficiency causes a childhood onset cerebellar degeneration syndrome clinically defined as SCAR20 and characterized by cerebellar ataxia and intellectual disability. Previous work identified lysosome- and autophagy-specific defects in cultured patient neural progenitor–like cells (16, 17), and recent evidence implicates SNX14 in LD biogenesis, FA desaturation, and nonvesicular interorganelle lipid transport (19, 22–24). However, most of these studies were performed in cultured cells with unclear relevance for SCAR20 pathology. To overcome this limitation and study pathogenic mechanisms that selectively affect the cerebellum, we generated a *Snx14*-KO mouse that closely recapitulates SCAR20 at genetic and phenotypic level. Consistent with a widespread expression of SNX14, we find that SNX14 deficiency in vivo leads to tissue-specific lipid metabolite and storage defects that likely result from cell type–specific lipid homeostatic requirements. Remarkably, predegenerating *Snx14*-KO cerebella is distinguishable from nondegenerating cerebral cortex by a unique accumulation of AcCa and L-carnitine, as well as depletion of TG levels. These data, combined with reduced LD numbers and enlarged telolysosomes in predegenerating PCs, suggest that lipid homeostasis defects cause cerebellar degeneration in SNX14 deficiency. However, due to lack of cellular resolution, our data do not rule out the possibility that other cell types in the cerebellum may also contribute to the lipidomic changes and neurodegeneration in *Snx14*-KO cerebella.

Lipid homeostasis disruption is associated with many cerebellar neurodegenerative disorders (8). Little is known, however, about the mechanisms that preserve lipid homeostasis in the cerebellum. PCs are fast and high-frequency spiking neurons with a large membrane area, which makes them particularly susceptible to oxidative stress induced membrane lipid peroxidation (41). Portions of membranes that contain peroxidated lipids are often cleared by autophagy, which leads to an overproduction of FAs and their storage in LDs as a protective mechanism from excess FA induced damage (39, 42, 43). Although neurons produce few LDs, recent evidence implicates autolysosome-derived structures in the clearance of toxic lipids through exocytosis in neurons (44). Therefore, our data showing abnormal lipidomic profiles and lipid storage and clearance organelles in predegenerating cerebella fit with a model implicating SNX14 in the storage (LD) and clearance (lysosome) of toxic lipids generated in PCs. Notably, SNX14 has been associated with lysosome function regulation (16), and recent structural predictions suggest a role in interorganelle lipid transport (24) that may be important for PC-specific lipid homeostasis. Furthermore, lipid clearance and storage defects have recently been associated with neuronal ferroptosis (44), and our transcriptomics data show upregulation of ferroptosis-associated genes (i.e., *Dcn* and *Fabp5*) in predegenerating *Snx14*-KO cerebella and genes associated with iron at older ages. These data suggest an exciting hypothesis implicating lipotoxicity-induced ferroptosis as a pathogenic mechanism of cerebellar degeneration in SCAR20 that warrants future investigation.

Given the widespread expression of *Snx14*, it is possible that other cell types contribute to PC degeneration. Indeed, glia cells have a central role in the clearance and metabolism of neuronal lipids (44–46). Remarkably, loss of PCs in *Snx14*-KO cerebella overlaps with a robust gliosis. Given the enrichment of *Snx14* expression in cerebellar PCs reported in the literature and our RNAscope analyses, we predict that PC degeneration is primary to SNX14 deficiency, which then triggers gliosis in *Snx14*-KO cerebella. In agreement, the predegenerating *Snx14*-KO cerebellar transcriptomics data show an upregulation of *Dcn*, which encodes a protein that stimulates the immune response after being released by cells dying from ferroptosis (34). Recent reports also suggest that gliosis is induced by PC degeneration in cerebellar ataxias. For example, PC-specific expression of mutant *ataxin1* in Sca1154Q/2Q mice is enough to induce astrogliosis and microgliosis (47) and deletion of mutant *ataxin7* from PCs prevents gliosis in SCA7-92Q BAC mice (48). Future studies will investigate if the loss of SNX14 affects lipid homeostasis in glia and whether this contributes to the selective cerebellar degeneration in SNX14 deficiency.

Similar to recently reported SNX14-deficient mice (25, 49), the homozygous 1 bp deletion in our *Snx14*-KO mice causes loss of full-length SNX14 protein and low RNA counts across all coding exons. Unlike previous models that showed fully penetrant embryonic lethality (25, 49), about one-third of our *Snx14*-KO mice developed and survived to adulthood with a phenotype that resembles SCAR20. This finding suggests that SNX14 deficiency in humans may also interrupt embryonic development and cause SCAR20 only when embryonic lethality is circumvented. Although we still do not know what factors determine the developmental success or failure in SNX14 deficiency, there is a striking difference in the genetic architecture of *Snx14* mutations between organisms that show full and partial embryonic lethality. For instance, SNX14-deficient mice that completely fail to develop carry deletions of at least 1 full exon while SCAR20 patients and animal models — including our *Snx14*-KO mice, dogs (50), and zebrafish (25) — carry truncating point mutations or small indels. This observation has interesting implications for the generation of animal models of human disorders and for the pathogenic prediction of truncating genetic mutations that warrant further investigation.

Another factor that can influence the outcome of SNX14-deficient embryos is the environment and diet lipid composition. In line with this idea, SNX14-deficient cells are more vulnerable than control cells to saturated FAs (25), and treatment with valproic acid, a branched short-chain FA, partially rescued PC degeneration in a conditional mouse model (49). Furthermore, previous studies have shown that maternal diet lipid composition can modulate brain lipidome either embryonically by maternal feeding or in adult mice (37). Altogether, these data open a window to alter the course of SCAR20 through therapeutic diets. In this regard, further elucidating mechanisms that preserve lipid homeostasis in neurons, and particularly in the cerebellum, is of crucial relevance.

Overall, our work highlights the relevance of lipid homeostasis for neurodegenerative disorders and suggests a mechanism for increased susceptibility of the cerebellum to the expanding class of disorders caused by disrupted lipid metabolism pathways. Furthermore, our study provides a mouse model and molecular targets for future therapeutic studies.

## Methods

Detailed materials and methods are available in Supplemental Methods.

### Sex as a biological variable

Our study examined male and female animals, and similar findings are reported for both sexes.

### Animals

#### Generation of mouse model.

*Snx14*-KO mice were generated by pronuclear injection of 5 ng/μL Cas9 mRNA and 2.5ng/μL sgRNA (5′-GTAAACACGTTCTCCAAC-3′) in 1 cell–stage fertilized embryos obtained from superovulated C57BL/6J females mated with C57BL/6J males. Pups carrying *Snx14* indel alleles were selected for backcross with WT C57BL/6J mice for 3–6 generations (to filter out potential off targets) and further expanded as an experimental model. Only the *Snx14* c.1432delG carriers generated homozygous pups.

### Behavior analysis

#### Experimental design.

Behavior analysis was performed with 3 cohorts of WT and *Snx14*-KO littermates starting at 8 months of age. Each cohort contained mixed genotype and sex of animals. Behavior tests were performed in the following order: accelerating rotarod, catwalk, Metz ladder, and social choice and recall. Investigators were blinded to results during scoring of behavioral assessments. Whenever possible, offline analysis by computer software was utilized to enhance rigor.

#### Accelerating rotarod.

On day 1, mice were habituated to the stationary rotarod for 2 minutes. This was immediately followed by a trial where rotation was programmed to rise from 4 to 40 rpm in 300 seconds. After a 30-minute intertrial interval (ITI), a second trial was performed, followed by another ITI and a third trial. Three additional trials were performed on the next 2 consecutive days, for a total of 9 trials. A trial was terminated when a mouse fell, made 1 complete revolution while hanging onto the rod, or after 300 seconds. Latency to fall (time stayed until falling or riding the rod for a single revolution) was determined. Learning rate was calculated as followed: learning rate = (Trial 9 latency to fall — Trial 1 latency to fall)/8, where 8 is the number of intertrial intervals in this study.

#### Catwalk gait analysis.

In the catwalk gait analysis assay, mice were placed on a meter-long illuminated glass plate walkway in a dark room. A high-speed video camera below the plate recorded the paw prints, as the mice traversed a 20 cm section of the alley. The paw print footage was analyzed by CatWalk XT program (Noldus).

#### Metz ladder rung waking test.

The Metz procedure used a 1-meter–long horizontal ladder, which was about 1 cm wider than the mice. The Plexiglas walls were drilled with 3 mm holes to accept the metal rungs. The gaps between the rungs were randomly spaced 1–5 cm apart so that the mice had to adjust the projection of the landing of each paw. Mice were trained to run the ladder with all rungs in place, 1 cm apart before the test trials began. In the test, each mouse was placed at the beginning of the ladder. Five trials were performed on consecutive days and videotaped. The pattern of the rungs was changed after each trial to prevent animals from adapting. Trials were recorded by a high-definition digital camera. Foot slips of each trial were quantified later by an investigator blinded to group designation with video.

#### Social choice and recall test.

Mice were tested for social preference and recall as described previously (51). The testing apparatus was a rectangular Plexiglas 3 chamber arena (60 × 40 × 20 cm [L × W × H]). The chamber was continuous with areas at the ends designated for the placement of vented cylinders to hold the cues. The social cues were juvenile, sex-matched C57BL/6J mice. The inanimate cues were smooth rocks that approximate the size of the social cues. The procedure consisted of a habituation phase whereby the experimental mouse was placed into the center chamber with empty cylinders in the side chambers for 10 minutes. After habituation, the choice phase immediately began. The cylinders were loaded with either a social cue (young mouse, M1) or inanimate cue. The experimental mouse was allowed to explore the cues for 10 minutes. Immediately after the choice phase, the recall phase was performed. The now familiar social cue, M1, remained in a cylinder while a novel mouse, M2, was loaded into the cylinder that previously held the inanimate cue. The experimental mouse was allowed to freely explore the 2 social cues for 10 minutes. The bouts and duration of explorations (nose ≤ 1 cm proximity) with the cylinders was determined with ANYmaze software (Stoelting Co.).

### Histology

#### IF staining.

Mice were anesthetized with isoflurane (Terrell) and perfused transcardially with 20 mL 1× PBS and 20 mL 4% paraformaldehyde (PFA) (Electron Microscopy Sciences). Brains dissected out from scalp were postfixed in 4% PFA for 18 hours at room temperature (RT) and washed 3 × 10 minutes in 1× PBS. Brains were sliced into 50 μm sections using a vibratome (Leica).

On the day of staining, slides were washed with 1× PBS, permeabilized, and blocked with PBS + 0.3% Triton X-100 (PBST) and 4% goat serum (G9023, Sigma-Aldrich) for 45 minutes at RT. Slides were then incubated with primary antibodies (Supplemental Methods) in 2% goat serum in PBST at 4°C on the shaker overnight. The next day, slides were washed with PBST 3 × 10 minutes and incubated with Alexa Fluor–conjugated secondary antibodies at 1:500 in 2% normal goat serum in PBST for 2 hours at RT. Slides were washed in PBST 3 × 10 minutes, incubated with 300 nM DAPI (D3571, Invitrogen) for 10 minutes at RT, and mounted on microscope slides with ProLong Gold antifade (P36930, Invitrogen) or Mowiol (81381, MilliporeSigma) covered with a coverslip. Immunostainings were imaged with a Leica TCS SP8 X confocal microscope, and images were processed and quantified with ImageJ (NIH).

#### RNAscope in situ hybridization.

The RNAscope in situ hybridization was performed as recommended by the manufacturer with reagents from Advanced Cell Diagnostic (Supplemental Methods). Once RNAscope was completed, immunofluorescence staining was immediately performed as described above. Sections were imaged with a Leica TCS SP8 X confocal microscope, and images were processed and quantified with ImageJ.

#### BODIPY staining.

Fixed brain and liver tissue was sliced into 50 μm sections using vibratome, rinsed in PBS, and incubated with 2 μM BD493 (D3922, Invitrogen) for 30 minutes at RT with gentle rocking. Then, the sections were rinsed in PBS 3 × 10 minutes and mounted on microscope slides with Mowiol and covered with coverslips.

#### TEM.

Mice were perfused with 20 mL of PBS, followed by 20 mL 2% PFA and 2% glutaraldehyde in sodium cacodylate buffer. Cerebella were dissected, trimmed to 1 mm thickness, and processed for TEM at the University of Delaware’s Bio-Imaging Center (Newark, Delaware, USA). Briefly, tissues were washed 3 × 15 minutes in 0.1M sodium cacodylate buffer pH 7.4 and postfixed for 2 hours with freshly prepared 1% osmium tetroxide and 1.5% potassium ferrocyanide in 0.1M sodium cacodylate buffer (pH 7.4) or — alternatively, to improve LD detection — with 1% osmium tetroxide in 0.1M imidazole pH 7.5. The tissue was washed with water, dehydrated through an ascending acetone series, and then infiltrated with Embed-812 resin. The next day, samples were embedded in flat-bottom capsules and polymerized at 60°C overnight. Ultrathin sections were cut using a Leica UC7 ultramicrotome and placed onto single hole 1,500 μm copper aperture grids with a formvar/carbon film. Sections were poststained with 2% uranyl acetate in 50% methanol (MeOH) and Reynolds’ lead citrate and examined on a Thermo Fisher Scientific Talos L120C transmission electron microscope operating at 120 kV. Images were acquired with a Thermo Fisher Scientific Ceta 16M camera. Quantification of area and numbers was done by ImageJ.

#### Matrix-assisted laser desorption ionization coupled to TOF (MALDI-TOF) MS imaging.

MALDI-TOF MS imaging was carried out in MALDI MS Imaging Joint Facility at Advanced Science Research Center of City University of New York Graduate Center.

Eight-week-old mouse brains were cryosectioned (10 μm thickness) sagittally and gently transferred onto the precooled conductive side of indium tin oxide–coated (ITO-coated) glass slides (Bruker Daltonics) for MALDI imaging. Mounted cryosections were desiccated in vacuum for 45 minutes at RT, followed by matrix deposition using HTX M5 sprayer (HTX Technologies) on DHB matrix (40 mg/mL in MeOH/water [70/30, v/v], flow rate of 0.05 mL/min, and a nozzle temperature of 85°C for 8 cycles) to detect metabolites and lipids. MALDI mass spectra were acquired in positive ion mode (DHB) acquired by MALDI-TOF MS Autoflex (Bruker Daltonics). MALDI imaging data were recorded and processed using FlexImaging v3.0 and were further analyzed using SCiLS (2015b version). Ion images were generated with root-mean square (RMS) normalization and a bin width of ± 0.15 Da. The spectra were interpreted manually, and analyte assignment was achieved by comparing with LC-MS/MS experiment results (52). The signal intensity of the cortex and cerebellum regions of 3 animals of each genotype were quantified using SCiLS and were further analyzed using GraphPad. *P* values between control and mutant animals were analyzed by two-tailed Student’s *t* test using 3 animals of each group.

### Cell Culture

#### PC culture.

Primary mixed cerebellar cultures were generated and maintained as described (53). Briefly, cerebellums were isolated from E16.5 of WT or *Snx14-*KO mice, dissociated, and plated at 50,000 cells on coverslips coated with 0.1 mg/mL poly-D-lysine in recovery media — DMEM/F-12, (11330032, Thermo Fisher Scientific) supplemented with 1% penicillin-streptomycin (15140122, Thermo Fisher Scientific), 1X B-27 (17504044, Thermo Fisher Scientific), 10% FBS (101, Tissue Culture Biologicals), 20 μg/mL insulin (I9278, MilliporeSigma), and 100 ug/mL IGF-1 (100-11, PeproTech). Two hours later, recovery media were removed and replaced with complete media (DMEM/F-12, supplemented with 1% penicillin-streptomycin, 1X B-27, 1% FBS, 20 μg/mL insulin, and 100 ug/mL IGF-1). PCs were cultured for 7 days in vitro before processing for experiments.

#### PC LD analysis.

To promote LD biogenesis, cerebellar cultures were incubated with 600 uM OA (O1008, MilliporeSigma) conjugated to 100 uM FA-free BSA (A1595, MilliporeSigma) overnight. Cells were fixed 10 minutes with 4% PFA at RT, washed with 1× PBS, and blocked in blocking buffer (1.5% glycine, 3% BSA, 0.01% saponin in 1× PBS) for 1 hour at RT. Cells were incubated overnight at 4°C with primary antibodies in antibody solution (1% BSA, 0.01% saponin in 1× PBS). The following day, cells were washed, incubated in secondary antibodies with 300 nM DAPI and 2 μM BD493 for 2 hours at RT, and mounted with Fluoromount-G (00-4958-02, Invitrogen). Images for quantification were captured with Leica TCS SP8 X confocal microscope and BD493^+^ puncta quantified with the “analyze particles” plug-in in Fiji-ImageJ after processing with “Intermodes” algorithm.

### Biochemical studies

#### WB.

Mouse tissue was dissected, fast-frozen, and stored in –80°C until use. On the experiment day, tissue was homogenized in RIPA buffer (9806, Cell Signaling) supplemented with a protease inhibitor cocktail (P8340, Sigma-Aldrich) and incubated for 15 minutes at 4°C. After samples were centrifuged at 16,400*g* at 4°C for 15 minutes, supernatant containing protein extract was collected, mixed with 1× LDS loading buffer (B0007, Invitrogen) supplemented with 200 mM DTT (BP172-5, Thermo Fisher Scientific), and loaded on a 4%–15% Mini-Protean TGX Precast Protein Gel. Proteins were transferred onto PVDF membranes in Mini Gel Tank at 80V for 180 minutes. Membranes were blocked with 5% milk-TBST or EveryBlot Blocking Buffer (12010020, Bio-Rad) for 1 hour at RT and then probed with primary antibodies diluted in 5% milk-TBST or EveryBlot Blocking Buffer solution overnight at 4°C. The next day, membranes were washed and probed with horseradish-peroxidase–conjugated secondary antibodies for 1 hour at RT, incubated in either Pierce ECL Western Blotting Substrate kit (32106, Thermo Fisher Scientific) or SuperSignal west dura extended duration substrate (34076, Invitrogen), and exposed on autoradiography film following development in AFP Mini-Med 90 X-Ray Fil Processor. Exposed films were scanned, and protein bands were quantified using ImageJ.

#### RNA-Seq.

One-month-old or 1-year-old mice were euthanized, and tissue was dissected on ice, fast frozen, and stored in –80°C until RNA extraction. Total RNA from the cerebellum or cerebral cortex were isolated using TRIzol (15596026, Invitrogen) reagent according to the manufacturer’s instructions. Strand-specific mRNA-Seq libraries for the Illumina platform were generated and sequenced at GENEWIZ or Novogene following the manufacturer’s protocol with sample-specific barcodes for pooled sequencing. After sequencing in Illumina HiSeq or Novoseq platform with 2× 150 PE configuration at an average of 15 million reads per sample, sequenced reads were trimmed to remove possible adapter sequences and poor-quality nucleotides, and trimmed reads were mapped to the Mus musculus GRCm38 reference genome using Spliced Transcripts Alignment to a Reference (STAR v2.7.3a) software. Reads were counted using FeatureCounts from the subread package (v2.0.1) (54). Transcripts per million (TPM) values were calculated from featureCounts-derived counts. Heatmap of gene expression were generated using the tidyverse R package with *Z* score of the log_2_(tpm+1). Differential gene expression analysis was performed with DEseq2 (v1.38.3). Raw *P* values were adjusted using the Benjamini-Hochberg method. DEGs were defined as having an *P*_adj_ < 0.05. Volcano plots were generated with the EnhancedVolcano R package. Functional enrichment analysis was conducted utilizing the enrichR R package. GSEA was performed on the *Mus musculus* msigdbr database in the C5 ontology category. Relevant lipid, oxygen, or iron-related terms were manually selected and displayed in the waterfall plots, generated through the tidyverse R package.

### UPLC-HRMS whole lipidome analysis

#### Sample preparation.

Two-month-old mice were euthanized and, after heart blood collection, cortex, cerebellum, and liver were dissected, these organs were snap-frozen in liquid nitrogen and stored at –80°C until lipid extraction. For lipid extraction, plasma samples were prepared as previously reported (55). About 10 mg of frozen tissue fragments were weighted, chopped, and mixed with 0.6 mL 80% MeOH and 10 μL on internal standard mix (SPLASH LIPIDOMIX, 330707, Avanti Polar Lipids). Samples were pulse sonicated in ice for 30× 0.5 seconds, incubated for additional 20 minutes in ice, and vortexed 3× 30 seconds, and tissue homogenates were transferred to a 10 mL glass Pyrex tube with screw cap. Then, 5 mL methyl tert-butyl ether (MTBE) was added to each tube and vigorously shacken for 30 minutes, followed by the addition of 1.2 mL water and a 30-second vortex. Samples were centrifuged for 10 minutes at 1,000*g* at RT, and the top clear phase was collected to a clean glass Pyrex tube and dried down under nitrogen. For the analysis, dried samples were resuspended in 100 μL MTBE/MeOH = 1/3 (v/v), spun down at 10,000*g* for 10 minutes at 4°C. The top 50 μL were transferred to a HPLC vial, and 2 μL were injected for LC-MS analysis.

#### LC-HRMS for lipids.

Separations were conducted on an Ultimate 3000 (Thermo Fisher Scientific) using an Ascentis Express C18, 2.1 × 150 mm 2.7 μm column (Sigma-Aldrich). For the HRMS analysis, a recently calibrated QE Exactive-HF mass spectrometer (Thermo Fisher Scientific) was used in positive ion mode with an HESI source. Control extraction blanks were made in the same way using just the solvents instead of the tissue homogenate. Untargeted analysis and targeted peak integration was conducted using LipidsSearch 4.2 (Thermo Fisher Scientific) as described by Wang et al. (56). Lipid quantification was done from the full-scan data. The areas were normalized based on the amount of the internal standard added for each class. All amounts were then normalized to the original tissue weight.

### Statistics

Statistical analyses were performed using GraphPad Prism 8 (GraphPad Software Inc.). When possible, data were analyzed by researchers blinded to the genotype. Sample size for each experiment was determined based on similar studies. To compare the means of groups in which normal distribution and similar variance between groups was confirmed, two-tailed Student’s *t* test (for 2 groups), 1-way ANOVA (for more than 2 groups), or 2-way ANOVA followed by Šidák’s or Tukey’s post hoc test (for multiple variables) was used. A *P* value less than 0.05 was considered statistically siginficant. Outliers were removed in 2 behavioral studies using the ROUT method with Q = 1%, *P* < 0.0002. To test the goodness of fit between an observed distribution and a theoretical distribution of frequencies, a chi-square test (χ^2^) was used. For differential gene expression analysis of RNA sequencing data, raw *P* values were adjusted using the Benjamini-Hochberg method

### Study approval

All animal procedures were performed according to the *Guide for the Care and Use of Laboratory Animals* (National Academies Press, 2011) and approved by the IACUC at Children’s Hospital of Philadelphia.

### Data availability

RNA-Seq data were deposited in GEO under the accession no. GSE215834. Whole data from lipidomic analysis are available in Supplemental Data 3. All other data are available in the Supporting Data Values file.

## Author contributions

Study conceptualization and design were contributed by YZ, VS, and NA. Validation and maintenance of mouse colony were contributed by YZ, TJ, and NA. Behavioral study design, execution, and data collection were contributed by BC and TO. Behavioral data analysis was contributed by BC, TO, YZ, and HT. Histology, cell culture, and TEM studies were contributed by YZ, VS, MF, and DY. RNA extraction and RNA-Seq analysis were contributed by YZ. Lipidomic analysis was contributed by YZ, PX, and CM. MALDI MS imaging analysis was contributed by SL and YH. Data interpretation was contributed by YZ, VS, MH, and NA. Supervision and project administration were contributed by NA. Manuscript preparation was contributed by YZ, VS, and NA. Manuscript editing and review were contributed by all authors.

## Supplementary Material

Supplemental data

Supplemental data set 1

Supplemental data set 2

Unedited blot and gel images

Supporting data values

## Version 1 04/16/2024

In-Press Preview

## Version 2 05/22/2024

Electronic publication
