# Supplementary material for: Altered lipid homeostasis is associated with cerebellar neurodegeneration in SNX14 deficiency
Source: JCI Insight. 2024 Apr 16;9(10):e168594. doi: 10.1172/jci.insight.168594 (PMC11141923; doi:10.1172/jci.insight.168594)
Supplement: Supplemental data [file jciinsight-9-168594-s188.pdf]

Supplementary Materials for

# Altered lipid homeostasis is associated with cerebellar neurodegeneration in SNX14 deficiency

Yijing Zhou[1,2*], Vanessa B. Sanchez[1,2*], Peining Xu[3], Thomas Roule[1,2], Marco Flores-Mendez[1,2], Brianna Ciesielski[4], Donna Yoo[1,2], Hiab Teshome[1,2], Teresa Jimenez[1,2], Shibo Liu[5], Mike Henne[6], Tim O'Brien[4], Ye He[5,7], Clementina Mesaros[3], Naiara Akizu[1,2, #]

#Corresponding author. Email: aquizun@chop.edu

**This PDF file includes:**

Supplemental Methods
Supplemental Figures 1 through 7

**Supplemental Methods:**

***<u>Sex as a biological variable</u>***

Our study examined male and female animals, and similar findings are reported for both sexes.

***<u>Animals</u>***

***Generation of mouse model***

*Snx14* KO mice were generated by pronuclear injection of 5ng/ul Cas9 mRNA and 2.5ng/ul sgRNA (5'- GTAAACACGTTCTCCAAC-3') in 1 cell stage fertilized embryos obtained from superovulated C57BL/6J females mated with C57BL/6J males. Pups were genotyped by PCR with forward 5'-cctttctgttactcagcaataacttg-3', reverse 5'-tgaatttggaattgcgtgtg-3' primers, followed by Sanger sequencing and those carrying *Snx14* indel alleles selected for backcross with WT C57BL/6J mice for 3-6 generations (to filter out potential off targets) and further expanded as an experimental model. Only the Snx14 c.1432delG carriers generated homozygous pups.

***Animal maintenance and husbandry***

Mice were group-housed at a maximum 5 animals per cage with a 12-h light/dark cycle at constant temperature with ad libitum access to food and water and maintained in C57BL/6J background (C57BL/6J, Jackson Laboratories Stock No: #000664). To obtain homozygous *Snx14* KO mice and WT littermates, heterozygous male and female mice were mated. Genotyping was performed by qPCR following Transnetyx Inc. (Cordova, TN) custom protocol with DNA obtained from tail clips.

***Behavior analysis***

***Experimental design***

Behavior analysis was performed with three cohorts of WT and *Snx14* KO littermates starting at 8 months of age. Each cohort contained mixed genotype and sex of animals. Behavior tests were performed in the following order: accelerating Rotarod, Catwalk, Metz Ladder and Social choice/recall. Investigators were blinded during scoring of behavioral assessments. Whenever possible, offline analysis by computer software was utilized to enhance rigor.

***Accelerating Rotarod***

On day 1, mice were habituated to the stationary Rotarod for 2 minutes. This was immediately followed by a trial where rotation was programmed to rise from 4-40rpm in 300 seconds. After a 30-minute intertrial interval (ITI), a second trial was performed, followed by another ITI and third trials. Three additional trials were performed on the next 2 consecutive days, for a total of 9 trials. A trial was terminated when a mouse fell, made one complete revolution while hanging onto the rod, or after 300s. Latency to fail (time stayed until falling or riding the rod for a single revolution) was determined. Learning rate was calculated as followed: learning rate = (Trial 9 latency to fall – Trial 1 latency to fall)/8, 8 is the number of inter-trial intervals in this study.

***Catwalk gait analysis***

In the Catwalk gait analysis assay, mice were placed on a meter-long illuminated glass plate walkway in a dark room. A high-speed video camera below the plate recorded the paw prints,

as the mice traversed a 20cm section of the alley. The paw print footage was analyzed by CatWalk XT program (Noldus, Leesburg, VA).

***Metz ladder rung waking test***

The Metz procedure used a 1-meter-long horizontal ladder, which was about 1cm wider than the mice. The Plexiglas walls were drilled with 3mm holes to accept the metal rungs. The gaps between the rungs were randomly spaced 1-5 cm apart so that the mice had to adjust the projection of the landing of each paw. Mice were trained to run the ladder with all rungs in place, 1cm apart before the test trials began. In the test, each mouse was placed at the beginning of the ladder. Five trials were performed on consecutive days and videotaped. The pattern of the rungs was changed after each trial to prevent animals from adapting. Trials were recorded by a high-definition digital camera. Foot slip(s) of each trial was quantified later by an investigator blinded to group designation with video.

***Social choice and recall test***

Mice were tested for social preference and recall as described previously (51). The testing apparatus was a rectangular Plexiglas three chamber arena (60 cm (L) × 40 cm (W) × 20 cm (H)). The chamber was continuous with areas at the ends designated for the placement of vented cylinders to hold the cues. The social cues were juvenile, sex-matched C57BL/6J mice. The inanimate cues were smooth rocks that approximate the size of the social cues. The procedure consisted of a habituation phase whereby the experimental mouse was placed into the center chamber with empty cylinders in the side chambers for 10 minutes. After habituation, the choice phase immediately began. The cylinders were loaded with either a social cue (young mouse, M1)

or inanimate cue. The experimental mouse was allowed to explore the cues for 10 minutes. Immediately after the choice phase, the recall phase was performed. The now familiar social cue, M1 remained in a cylinder while a novel mouse, M2 was loaded into the cylinder that previously held the inanimate cue. The experimental mouse was allowed to freely explore the 2 social cues for 10 min. The bouts and duration of explorations (nose ≤1 cm proximity) with the cylinderswas determined with ANYmaze software (Stoelting Co. Wood Dale Il.).

***Histology***

***Immunofluorescence staining***

Mice were anesthetized with isoflurane (Terrell) and perfused trans-cardially with 20ml 1X PBS and 20ml 4% paraformaldehyde (PFA) (Electron Microscopy Sciences). Brains dissected out from scalp were post-fixed in 4% paraformaldehyde for 18h in RT and washed 3 x 10 mins in 1X PBS. Brains were sliced into 50um sections using vibratome (Leica).

On the day of staining, slides were washed with 1X PBS, permeabilized and blocked with PBS+0.3% Triton X-100 (PBST) and 4% goat serum (G9023, Sigma-Aldrich) for 45 min at room temperature. Slides were then incubated with primary antibodies in 2% goat serum in PBST at 4˚C on the shaker overnight. Primary antibodies used include: Calbindin-1 (1:500, C9848, Sigma-Aldrich), GFAP (1:500, Z033429-2, Agilent), IBA1 (1:500, 019-19741, Wako), NeuN (1:500, MAB377, Sigma-Aldrich), and Lamp1 (1:200, 1D4B, DSHB). Next day, slides were washed with PBST 3 x 10 mins and incubated with Alexa Fluor-conjugated secondary antibodies at 1:500 in 2% normal goat serum in PBST for 2h at room temperature (RT). Secondary antibodies used include Alexa Fluor 488-labeled goat anti-mouse IgGs (A-11001, Invitrogen), Alexa Fluor 555-

labeled goat anti-mouse IgGs (A-21422, Invitrogen), Alexa Fluor 488-labeled goat anti-rabbit IgGs (A-11008, Invitrogen), and Alexa Fluor 555-labeled goat anti-rabbit IgGs (A-21428, Invitrogen). Slides were washed in PBST 3 x 10 mins, incubated with 300nM DAPI (D3571, Invitrogen) for 10min at RT and mounted on microscope slides with ProLong Gold antifade (P36930, Invitrogen) or Mowiol (#81381, Sigma) covered with a coverslip. Immunostainings were imaged with a Leica TCS SP8 X confocal microscope and images processed and quantified with ImageJ (NIH).

**RNAscope *In situ hybridization***

The reagents in the RNAscope Multiplex Fluorescent Reagent Kit v.2 (#323100), RNAscope Probe Diluent (#300041), HybEZ oven (#321710/321720), humidity control tray (#310012), HybEZ Humidifying Paper (#310025), ISH probe targeting Mm-*Snx14* (#895211), and Opal 520 (ASOP520) were all from Advanced Cell Diagnostic (USA). Brain tissue was processed as mentioned under Immunofluorescence staining. The RNAscope *in situ* hybridization was performed as recommended by the manufacturer. Briefly, *Snx14* WT and KO brain sections were washed once in 1X PBS, followed by three washes in 0.1% Triton X-100 and PBS, mounted on Superfrost Plus charged glass slides (#12-550-15, Thermo Fisher Scientific) and baked at 60 °C in the HybEZ oven for 25 min. The slides were then submerged in 4% PFA for 30 min and washed three times in $H_2O$. RNAscope $H_2O_2$ was applied to each section for 5 min at room temperature. The slides were then washed three times in $H_2O$ before being submerged in pre-warmed 90 °C $H_2O$ for 15 s, followed by pre-warmed 90 °C RNAscope Target Retrieval for 15 min. Slides were washed three times in $H_2O$ before RNAscope Protease III was applied to each section, and then incubated for 15 min at 40 °C in the HybEZ oven. Slides were washed three times in $H_2O$ and then

incubated with probe solution diluted to 1:50 with probe diluent for 2 h at 40 °C in the HybEZ oven. Next, the sections were washed three times in RNAscope wash buffer followed by fluorescence amplification, such that Opal 520 was used to mark the channel 1 probe, Mm-*Snx14*. Once RNAscope was completed, immunofluorescent staining immediately began such that the sections were incubated in blocking solution (4% normal goat serum in PBST) for 45 min at room temperature. Then sections were incubated in antibody solution (2% normal goat serum in PBST) with Calbindin-1 (1:500, C9848, Sigma-Aldrich) at 4 °C overnight. The next day, sections were washed three times with PBST 3 x 10 mins and then incubated with AlexaFluor 546 or 647 - conjugated donkey antibodies (Invitrogen, A31570 or A78947) in antibody solution for 2h at room temperature. Slides were washed in PBST 3 x 10 mins, incubated with 300nM DAPI (D3571, Invitrogen) for 10min at RT and mounted on microscope slides with ProLong Gold antifade (P36930, Invitrogen) or Mowiol (#81381, Sigma) covered with a coverslip. Sections were imaged with a Leica TCS SP8 X confocal microscope and images processed and quantified with ImageJ (NIH).

***BODIPY staining***

Mice were perfused as above, and brain and liver dissected out and post-fixed in 4% paraformaldehyde for 18h in RT following three washes with PBS. Tissue was sliced into 50µm sections using vibratome (Leica), rinsed in PBS and incubated with 2µM BODIPY 493/503 (Invitrogen D3922) for 30 min at RT with gentle rocking. Then, the sections were rinsed in PBS 3 x 10 mins and mounted on microscope slides with Mowiol (sigma #81381) covered with coverslips.

*Transmission Electron microscopy*

Mice were perfused with 20ml of PBS, followed by 20ml 2% paraformaldehyde and 2% glutaraldehyde in sodium cacodylate buffer. Then, cerebella were dissected out, trimmed to 1mm thickness, and processed for transmission electron microscopy at the University of Delaware's Bio-Imaging Center. Briefly, tissues were washed 3 x 15 min in 0.1M sodium cacodylate buffer pH 7.4 and post-fixed for 2 h with freshly prepared 1% osmium tetroxide and 1.5% potassium ferrocyanide in 0.1M sodium cacodylate buffer pH 7.4 or alternatively, to improve lipid droplet detection, with 1% osmium tetroxide in 0.1M imidazole pH 7.5. The tissue was washed 3 x 15 min with water. The samples were dehydrated through an ascending acetone series (25%, 50%, 75%, 95%, anhydrous 100%, anhydrous 100%), 15 min each step, and then infiltrated with Embed-812 resin (25% resin in acetone, 33% resin in acetone, 50% resin in acetone, 66% resin in acetone 75% resin in acetone) for 1 h each step. Following several changes in 100% resin, the tissue infiltrated overnight on a rotator, and the next day, samples were embedded in flat-bottom capsules and polymerized at 60°C overnight. Ultrathin sections were cut using a Leica UC7 ultramicrotome, and sections were placed onto single hole 1500-micron copper aperture grids with a formvar/carbon film. Sections were post-stained with 2% uranyl acetate in 50% methanol and Reynolds' lead citrate. Sections were examined on a ThermoFisher Scientific Talos L120C transmission electron microscope operating at 120kV, and images were acquired with a ThermoFisher Scientific Ceta 16M camera. Quantification of area and numbers was done by ImageJ (NIH).

***MALDI-TOF MS Imaging***

The Matrix-assisted laser desorption/ionization-time of flight (MALDI-TOF) mass spectrometry (MS) imaging was carried out in MALDI MS Imaging Joint Facility at Advanced Science Research Center of City University of New York Graduate Center. High purity grade 2,5-dihydroxybenzoic acid (DHB) matrix were purchased from TCI Chemicals, Phosphorus (red) were purchased from Millipore Sigma-Aldrich. Optima UHPLC/MS-grade methanol and water were purchased from Fisher Scientific.

Mouse brains were harvested at 8 weeks immediately after cervical dislocation and were snap-frozen for 5 min on an aluminum boat floating on liquid nitrogen. The frozen tissue was cryosectioned (10 μm thickness) using CryoStar NX50 (Thermo Scientific) at -18 °C (specimen head and chamber). Sagittal brain sections from the planes in a range from 2.2 mm to 3.2 mm off the middle line, corresponding to the plates 12-16 of Allen Mouse P56 Sagittal Brain Atlas, were collected and gently transferred onto the pre-cooled conductive side of indium tin oxide (ITO)-coated glass slides (Bruker Daltonics) for MALDI imaging. Mounted cryosections were desiccated in vacuum for 45 min at RT, followed by matrix deposition using HTX M5 sprayer (HTX Technologies, LLC). DHB (40 mg/mL in methanol/water (70/30, v/v), flow rate of 0.05 mL/min and a nozzle temperature of 85 °C for 8 cycles) were used to detect metabolites and lipids. MALDI imaging was performed on brain sections from 3 control brains and 3 SNX14 mutant brains and repeated twice. The following parameters were used for the DHB matrix: spray velocity of 1300 mm/min, track spacing of 2 mm, N2 gas pressure of 10 psi and flow rate of 3 L/min and nozzle height of 40 mm. MALDI mass spectra were acquired in positive ion mode (DHB) acquired by MALDI-TOF MS Autoflex (Bruker Daltonics). MS spectra were calibrated using red phosphorus as the standard for all experiments. Acquisition raster width set at 100 μm with laser smartbeam

parameter at ‘‘Medium’’. The imaging data for each array position were summed up by 500 shots at a laser repetition rate of 500 Hz. To minimize broadening of ion peaks, all the experiments were performed by setting laser power to its lowest value while allowing to accumulate ion spectra with appropriate S/N ratio. Spectra were acquired in the mass range from m/z 50 to 1300 Da with a low mass gate at 50 Da. MALDI imaging data were recorded and processed using FlexImaging v3.0, and further analyzed using SCiLS (2015b). Ion images were generated with root-mean square (RMS) normalization and a bin width of ± 0.15 Da. The spectra were interpreted manually, and analyte assignment was achieved by comparing with LC-MS/MS experiment results (52). The signal intensity of the cortex and cerebellum regions of three animals of each genotype were quantified using SCiLS and further analyzed using GraphPad. P-value between control and mutant animals were analyzed by Student’s t-test using three animals of each group.

## ***Cell culture***

### ***Purkinje Cell Culture***

Primary mixed cerebellar cultures were generated and maintained as described (53). Briefly, cerebellums were isolated from E16.5 of WT or *Snx14* KO mice, dissociated and plated at 50,000 cells on coverslips coated with 0.1 mg/mL poly-D-lysine in recovery media (DMEM/F-12, (#11330032, Gibco) supplemented with 1% Penicillin-Streptomycin (#15140122, Gibco), 1X B-27 (Gibco, # 17504044), 10% FBS (#101, Tissue Culture Biologicals), 20 ug/mL Insulin (#I9278, MilliPore Sigma), and 100 ug/mL IGF-1 (#100-11, PeproTech). Two hours later, recovery media was removed and replaced with complete media (DMEM/F-12, (#11330032, Gibco) supplemented with 1% Penicillin-Streptomycin (#15140122, Gibco), 1X B-27 (Gibco, # 17504044), 1% FBS (#101, Tissue Culture Biologicals), 20 ug/mL Insulin (#I9278, MilliPore

Sigma), and 100 ug/mL IGF-1 (#100-11, PeproTech). Purkinje cells were cultured for 7 days *in vitro* before processing for experiments.

***Purkinje Cell Lipid Droplet (LD) Analysis***

To promote LD biogenesis, cerebellar cultures were incubated with 600uM Oleic Acid (#O1008, Sigma) conjugated to 100uM fatty acid-free BSA (A1595, Sigma) overnight. Cells were then fixed with 4% PFA for 10 min at room temperature and blocked in blocking buffer (1.5% Glysine, 3% BSA, 0.01% Saponin in 1X PBS) for 1h at RT, and immunostained overnight at 4 °C with the antibody Calbindin-1 (1:500, C9848, Sigma-Aldrich) in antibody solution (1% BSA, 0.01% Saponin in 1X PBS) on a shaker overnight. After washing with 1X PBS for three times, secondary antibodies (AlexaFluor 546 or 647 -conjugated donkey antibodies (Invitrogen, A31570 or A78947) along with 300nM DAPI (D3571, Invitrogen) and 2μM BODIPY 493/503 (D3922, Invitrogen) diluted in antibody solution were added to the cells for 2h at room temperature. Finally, cells were washed with 1X PBS 3 times before mounting with Fluoromount-G (#00-4958-02, Invitrogen). Images for quantification were captured with Leica TCS SP8 X confocal microscope. Maximum intensity projection images of each channel for each image were made. Purkinje cells that were Calbindin-1-positive Purkinje cells were individually selected from these max projections and used for LD quantifications. Finally, BODIPY 493/503 signal was thresholded using the Fiji-ImageJ's "Intermodes" algorithm and the number of BODIPY 493/503-positive puncta were quantified using the "analyze particles" plug-in.

***Biochemical studies***

***Western blot***

Mouse tissue was dissected, fast-froze, and stored in -80˚C until use if needed. On the experiment day, tissue was homogenized in RIPA buffer (#9806, Cell Signaling) supplemented with a protease inhibitor cocktail (P8340, Sigma-Aldrich) with microtube homogenizers and incubated for 15 minutes at 4C. After centrifugation at 13,200 rpm, supernatant containing protein extract was collected, mixed with 1X LDS loading buffer (B0007, Invitrogen) supplemented with 200mM DTT (BP172-5, Fisher Scientific) and loaded on a 4-15% Mini-Protean TGX Precast Protein Gel. Proteins were transferred onto PVDF membranes in Mini Gel Tank at 80V for 180min. Membranes were blocked with 5% milk-TBST or EveryBlot Blocking Buffer (#12010020, Bio-Rad) for 1h at room temperature (RT) then probed with primary antibodies diluted in 5% milk-TBST or EveryBlot Blocking Buffer solution overnight at 4˚C. Primary antibodies used include: SNX14 (1:1,000, HPA017639, Sigma-Aldrich), Beta Actin (1:2,000, A00702, GenScript), LC3-B (1:1000, NB600-1384, Novus Biologicals), vGlut 1 (1:1000, #135311, SYSY), vGlut 2 (1:1000, #135042, SYSY), Synapsin (1:1000, #106103, SYSY), Synaptophysin (1:1000, MAB5258, Millipore Sigma), PSD-95 (1:1000, #516900, Invitrogen), and SV2A (1:1000, sc-376234, Santa Cruz Biotechnology). Membranes were then washed and probed with horseradish-peroxidase-conjugated donkey anti-mouse or anti-rabbit IgG (H+L) cross-adsorbed secondary antibody (SA1-100, Invitrogen; 31438, Invitrogen) for 1h at RT. Membranes were developed using either Pierce™ ECL Western Blotting Substrate kit (#32106, Thermo Scientific) or SuperSignal™ west dura extended duration substrate (34076, Invitrogen) and exposed on autoradiography film following development in AFP Mini-Med 90 X-Ray Fil Processor. Exposed films were scanned, and protein bands were quantified using ImageJ.

***RNA-seq***

1-month-old or 1-year-old mice were euthanized and tissue was dissected on ice, fast frozen, and stored in -80˚C until RNA extraction. On the day of RNA extraction, 50-100mg tissue from each sample was lysed in 1ml TRIzol (15596026, Invitrogen). Addition of 0.2ml chloroform (288306, Sigma-Aldreich) followed by centrifugation was performed to separate the solution into an aqueous phase and an organic phase. Aqueous phase was collected and Isopropyl alcohol was added to precipitate RNA. After centrifugation pellets were washed with 75% ethanol (111000200CSPP, Pharmco) and resuspended in RNAse free water. Strand-specific mRNA-seq libraries for the Illumina platform were generated and sequenced at GENEWIZ or Novogene following the manufacturer's protocol with sample specific barcodes for pooled sequencing. After sequencing in Illumina HiSeq or Novoseq platform with 2x150 PE configuration at an average of 15 million reads per sample, sequenced reads were trimmed to remove possible adapter sequences and poor quality nucleotides and trimmed reads mapped to the Mus musculus GRCm38 reference genome using Spliced Transcripts Alignment to a Reference (STAR v2.7.3a) software. Reads were counted using FeatureCounts from the subread package (v2.0.1)(54). TPM values were calculated from featureCounts-derived counts by normalizing the count rates to gene lengths and then scaling them by the sum of all normalized count rates, multiplied by $10^6$. Heatmap of gene expression was generated using the tidyverse R package with z-score of the log2(tpm+1). Differential gene expression analysis was performed with DEseq2 (v1.38.3) excluding genes with less than five reads, and all genes from chromosome X and Y. Differential expression was performed using a linear model and as factor the genotypes, for each time-point. Raw p-values were adjusted using the Benjamini-Hochberg method. Differentially expressed genes were defined as having an

adjusted p value of less than 0.05. PCA was conducted on variance-stabilized transformed counts using genotype, time and tissue as factor in the model to account for their effects on gene expression. Volcano plots of differentially expressed genes (DEGs) were generated with the EnhancedVolcano R package. Functional enrichment analysis was conducted utilizing the enrichR R package, incorporating only lists of DEGs containing five or more genes, and with databases: GO_Biological_Process_2023, GO_Cellular_Component_2023, GO_Molecular_Function_2023, Descartes_Cell_Types_and_Tissue_2021 and DisGeNET. Results were represented as dotplot with the size of each dot proportional to the fraction of significant DEGs relative to the total number of genes in each enriched term. Gene Set Enrichment Analysis (GSEA) was performed on the *Mus musculus* msigdbr database in the C5 ontology category. Relevant lipid, oxygen, or iron - related terms were manually selected and displayed in the waterfall plots, generated through the tidyverse R package. For inclusion in the waterfall visualization, terms were required to have a p-value less than 0.05.

***UPLC-HRMS whole lipidome analysis***

*Sample preparation*

1-2 mo mice were euthanized and, after heart blood collection, cortex, cerebellum, and liver were dissected and snap-frozen in liquid nitrogen. Blood, collected in lavender top tubes (RAM Scientific 07 6011), was centrifuged at 2000g for 15min at 4˚C and the supernatant (EDTA plasma) collected and snap-frozen in liquid nitrogen. Snap-frozen plasma and tissue were stored at -80 until lipid extraction. For lipid extraction, plasma samples were prepared as previously reported (55) and ~10mg of frozen tissue fragments were weighted and chopped with dry-ice-chilled blades on a chilled tile. The tissue was added to low retention Eppendorf tube filled with

0.6 mL 80% methanol (MeOH) and 10 µL on internal standard mix (SPLASH® LIPIDOMIX #330707, Avanti Polar Lipids) and kept in dry ice. Samples were pulse sonicated in ice for 30x 0.5 second and incubated for additional 20 min in ice for metabolite extraction. Each tube was then vortexed 3x 30 seconds and tissue homogenates transferred to a 10 mL glass Pyrex tube with screw cap. The Eppendorf tubes were rinsed with 0.5 mL methanol and added to corresponding glass Pyrex tube. Then, 5 mL methyl tert-butyl ether (MTBE) was added to each tube and vigorously shacken for 30 minutes, followed by the addition of 1.2 mL water and 30 second vortex. Samples were centrifuged for 10 min at 1000g to created two phases. The top clear phase was collected to a clean glass Pyrex tube and dried down under nitrogen. For the analysis, dried samples were resuspended in 100 µL MTBE/MeOH=1/3 (v/v), spun down at 10,000g for 10 min at 4°C. The top 50 µL were transferred to a HPLC vial and 2ul were injected for LC-MS analysis.

*Liquid chromatography high resolution -mass spectrometry (LC-HRMS) for lipids*

Separations were conducted on an Ultimate 3000 (Thermo Fisher Scientific) using an Ascentis Express C18, 2.1 × 150 mm 2.7µm column (Sigma-Aldrich, St. Louis, MO). Briefly, the flow-rate was 0.4 m/min, solvent A was water:acetonitrile (4:6 v/v) with 0.1% formic acid and 10 mM ammonium formate and solvent B was acetonitrile:isopropanol (1:9 v/v) with 0.1% formic acid and 10 mM ammonium formate. The gradient was as follows: 10 % B at 0 min, 10 % B at 1 min, 40 % B at 4 min, 75 % B at 12 min, 99 % B at 21 min, 99 % B at 24 min, 10 % B at 24.5 min, 10 % at 30 min. Separations were performed at 55 °C.

For the HRMS analysis, a recently calibrated QE Exactive-HF mass spectrometer (Thermo Fisher Scientific) was used in positive ion mode with an HESI source. The operating conditions were: spray voltage at 3.5 kV; capillary temperature at 285°C; auxiliary temperature 370°C; tube lens 45. Nitrogen was used as the sheath gas at 45 units, the auxiliary gas at 10 units and sweep

gas was 2 units. Same MS conditions were used in negative ionization mode, but with a spray voltage at 3.2 kV. Control extraction blanks were made in the same way using just the solvents instead of the tissue homogenate. The control blanks were used for the exclusion list with a threshold feature intensity set at 1e10^5. Untargeted analysis and targeted peak integration was conducted using LipidsSearch 4.2 (Thermo Fisher Scientific) as described by Wang et al (56).

An external mass calibration was performed using the standard calibration mixture approximately every three days. All samples were analyzed in a randomized order in full scan MS that alternated with MS2 of top 20, with HCD scans at 30, 45 or 60 eV. Full scan resolution was set to 120,000 in the scan range between m/z 250–1800. The pool sample was run every 15 samples. Lipids quantification was done from the full scan data. The areas were normalized based on the amount of the internal standard added for each class. All amounts were then normalized to the original tissue weight.

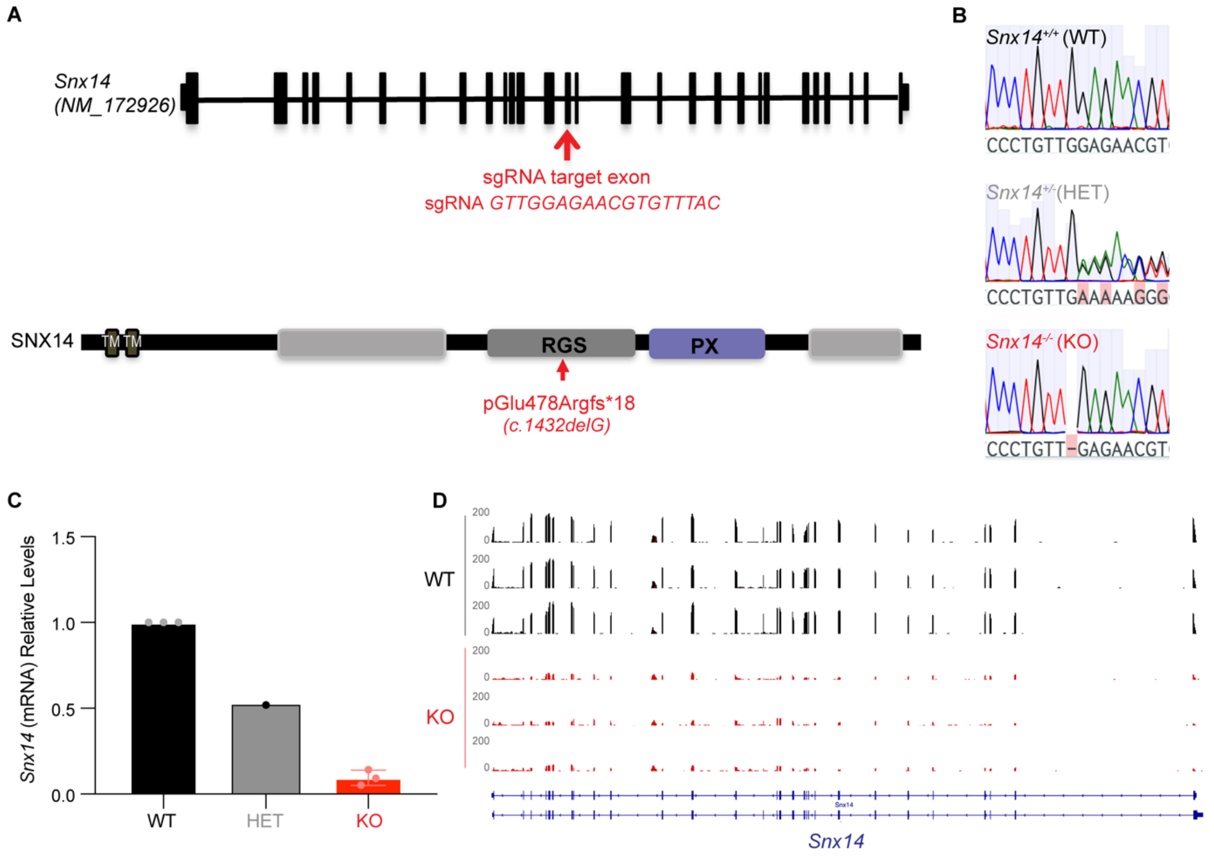


**Supplemental Figure 1. 1bp deletion in exon 14 of *Snx14* causes loss of SNX14 expression. (A)** Diagram showing the sgRNA sequence used to target exon14 with CRISPR/Cas9 (top) and the mutation carried by our *Snx14* KO mice. **(B)** Representative Sanger sequencing chromatograms of *Snx14*$^{+/+}$ (WT), *Snx14*$^{+/-}$ (HET), and *Snx14*$^{-/-}$ (KO) mouse PCR product, showing the c.1432delG homozygous deletion in a *Snx14* KO mouse. **(C)** RT-qPCR of *Snx14* in WT, HET, and KO mouse tissues, shows reduction of *Snx14* mRNA levels in KO mice. **(D)** Integrative Genomics Viewer (IGV) screenshot of RNAseq results showing less abundant read counts across all the *Snx14* exons in *Snx14* KO than in WT mice. Bottom blue diagrams show two alternative splice forms of *Snx14*. Thin lines represent introns and thick bars exons.

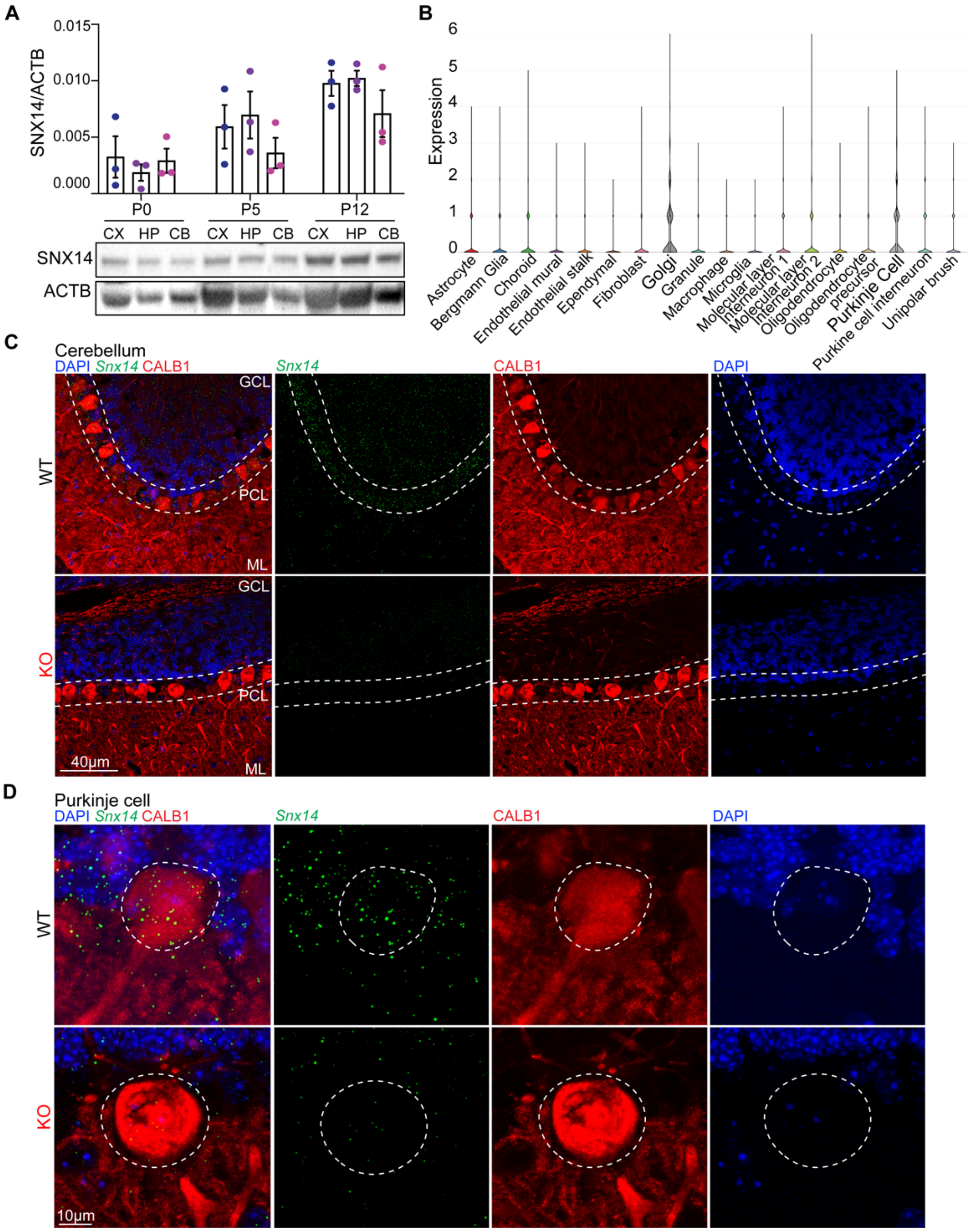


**Supplemental Figure 2. SNX14 is widely expressed in the brain and enriched in cerebellar Purkinje Cells (PCs).** (**A**) Western blot (WB) results showing the expression level of SNX14 in various brain regions in early postnatal ages (bottom). Graph shows relative SNX14 expression quantified by WB band densitometry of n=3 mice for each time point. CX, cortex; HP, hippocampus; CB, cerebellum. βeta-Actin (ACTB) is shown as loading control. (**B**) Violin plots showing distribution of cerebellar cells based on their *Snx14* expression levels. Data was extracted from cerebellar single nuclei (sn) RNAseq deposited in Broad Institute Single Cell Portal. (**C-D**) Representative *Snx14* RNAscope images of the cerebellar cortex (C) and a PC (D) in *Snx14* WT and KO mice, showing specific enrichment of *Snx14* mRNA in WT mouse PC layer. PCs are stained with anti-CALB1 antibody and DNA with DAPI.

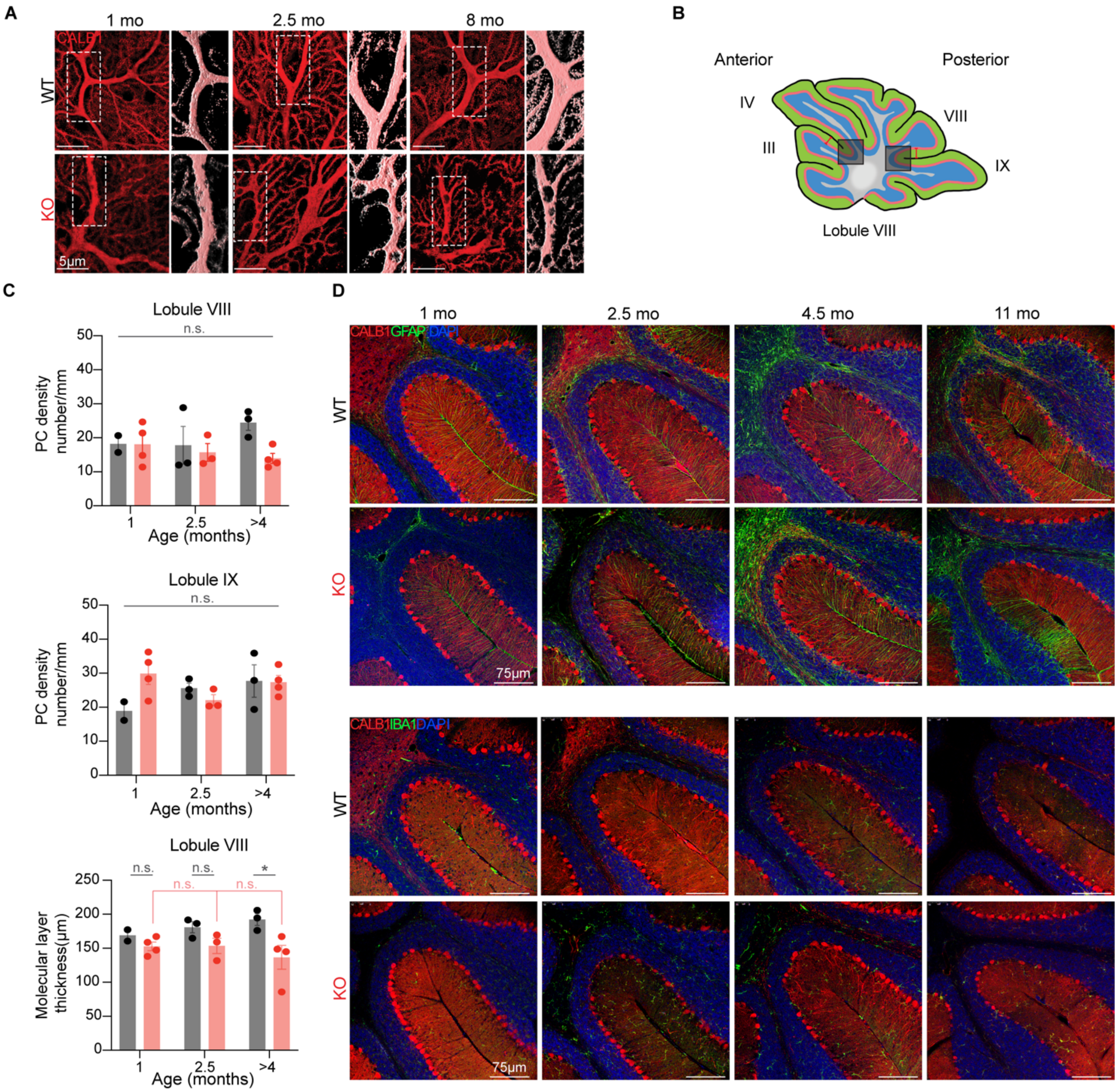


**Supplemental Figure 3. SNX14 deficiency causes late onset degeneration of posterior cerebella.** (**A**) Representative immunofluorescent staining of PCs with CALB1 antibody reveals progressive accumulation of vacuoles in *Snx14* KO mice PC dendrites. (**B**) Diagram showing cerebellar regions depicted in Fig 3D, G, H and Fig S3D, (**C**) PC density number quantified from Lobule VIII and IX, and molecular layer thickness quantified from Lobule VIII. 1mon: n=3 WT, n=4 KO; 2.5mon: n=3 WT, n=3 KO; >4mon: n=3 WT, n=4 KO. Two-way ANOVA followed by Sidak's multiple comparison test. (**D**) Representative immunofluorescent staining labeling Purkinje cells in red with anti-CALB1 antibody and astrocytes in green with anti-GFAP antibody (top) and microglia in green with anti-IBA1 antibody (bottom) at the base of Lobule VIII & IX. n.s. = non-significant, *$P$ <0.05.

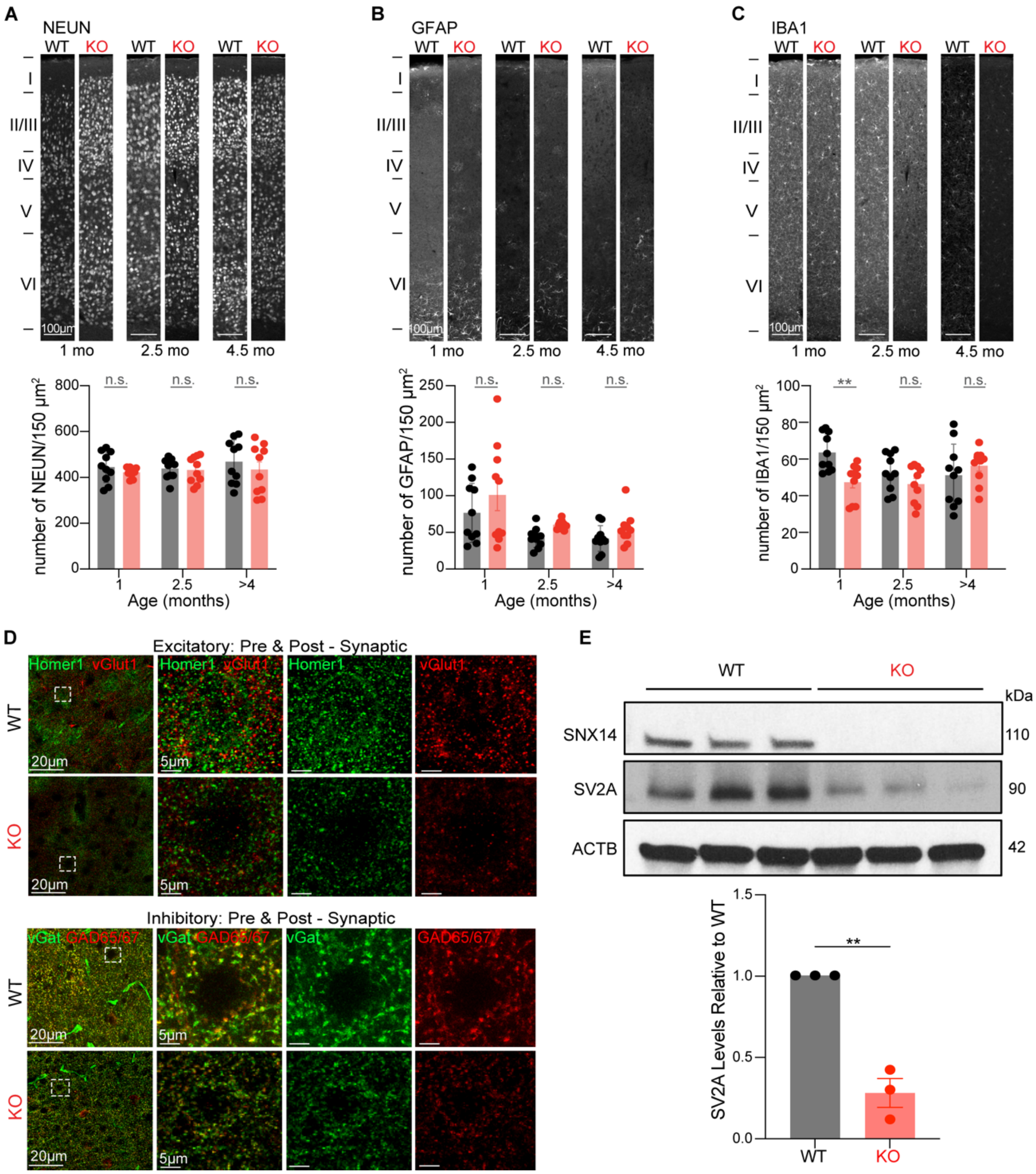


**Supplemental Figure 4. *Snx14* KO cerebral cortex shows defects in synaptic signaling without neurodegeneration.** (**A-C**) Coronal sections of cerebral cortices immunostained with (A) anti-NeuN to label neurons, (B) anti-GFAP to label astrocytes, and (C) anti-IBA1 to label microglia. Bar graphs showing average ±S.E.M of cell numbers per 150 µm$^2$ area in 4-5 regions of 3 mice per genotype and age. Two-way ANOVA followed by Sidak's multiple comparison test. (**D**) Representative immunofluorescence of markers for excitatory and inhibitory pre- and post-synaptic puncta in layer II/III of 1 year old *Snx14* WT and KO cerebral cortex sections. (**E**) Western Blot analysis of SV2A synaptic vesicle protein in three WT mice and their corresponding KO littermates at 1 year of age. ACTB is shown as loading control and SNX14 as proof of genotype identity. Bar graphs show mean ±S.E.M of relative SV2A/ACTB levels quantified by band densitometry analysis in n=3WT and n=3KO mice. Two-tailed student's *t*-test. n.s. = non-significant, $^{**}P < 0.01$.

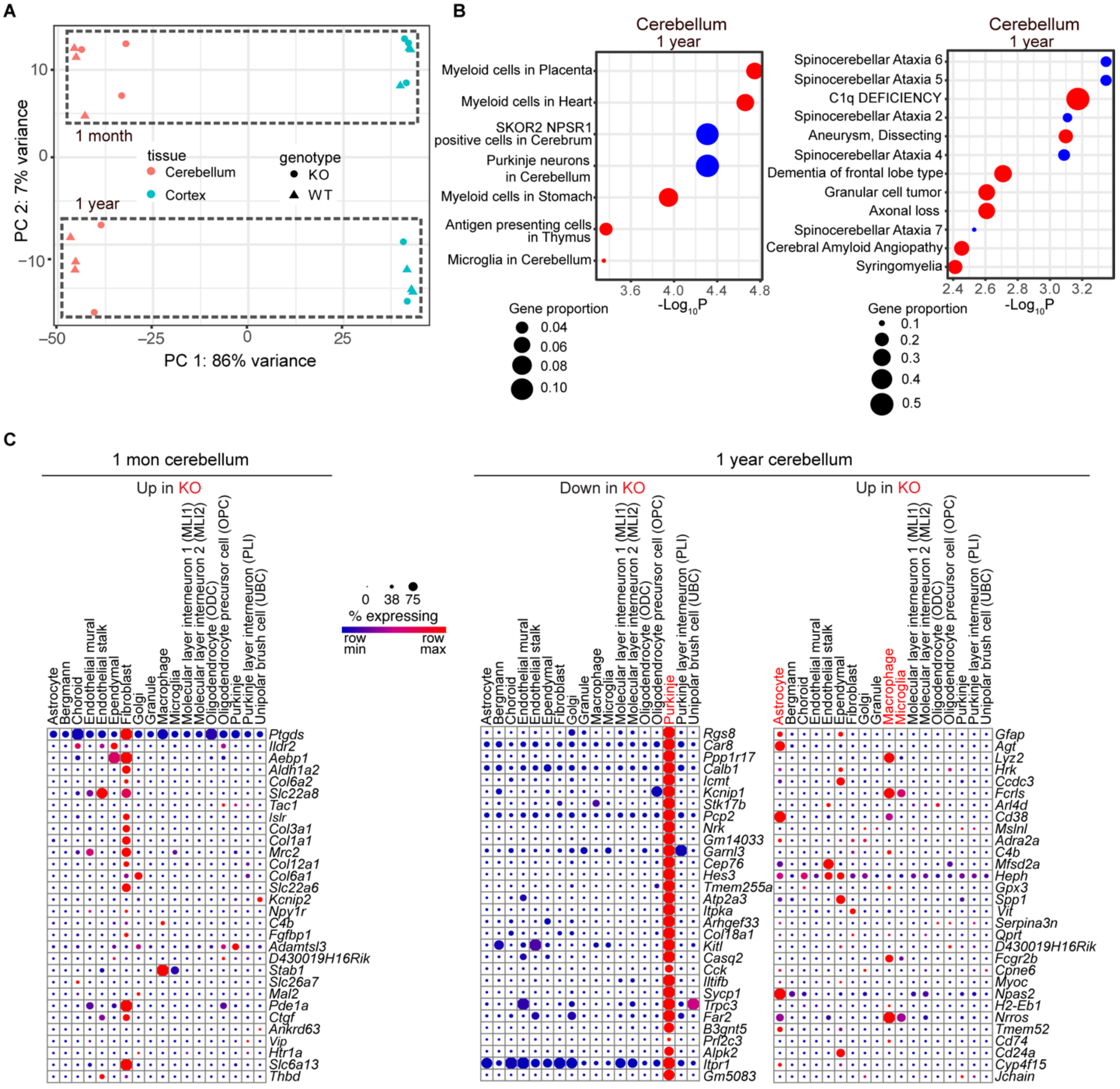


**Supplemental Figure 5. Loss of PC specific gene expression in 1-year-old *Snx14* KO cerebella. (A)** Principal component analysis (PCA) clustering of RNA-seq data from Cortex and Cerebellum in WT and KO mice at 1 month and 1 year, shows that the variance between brain regions contributes to the major difference, followed by age of animals. Samples are only clustered by genotype for cerebellum. **(B)** Dot plots of functional analysis using the Descartes Cell Types (left) and DisGeNET (right) databases of DEGs in 1-year Cerebellum, with down- and upregulated genes marked in blue and red, respectively. (**C**) Dot plot of cerebellar snRNAseq data *(30)* showing the average expression level (in blue to red scale) and percentage of cells (dot size) expressing the top 30 upregulated DEGs in 1 month old *Snx14* KO cerebella (from our bulk RNAseq results) (left) and for the top 30 downregulated DEGs (middle), and top 30 upregulated DEGs (right) in 1-year-old *Snx14* KO cerebella. Data shows that downregulated DEGs in 1-year-old *Snx14* KO are typically expressed in PCs which indicates that there is a loss of PCs in *Snx14* KO cerebella.

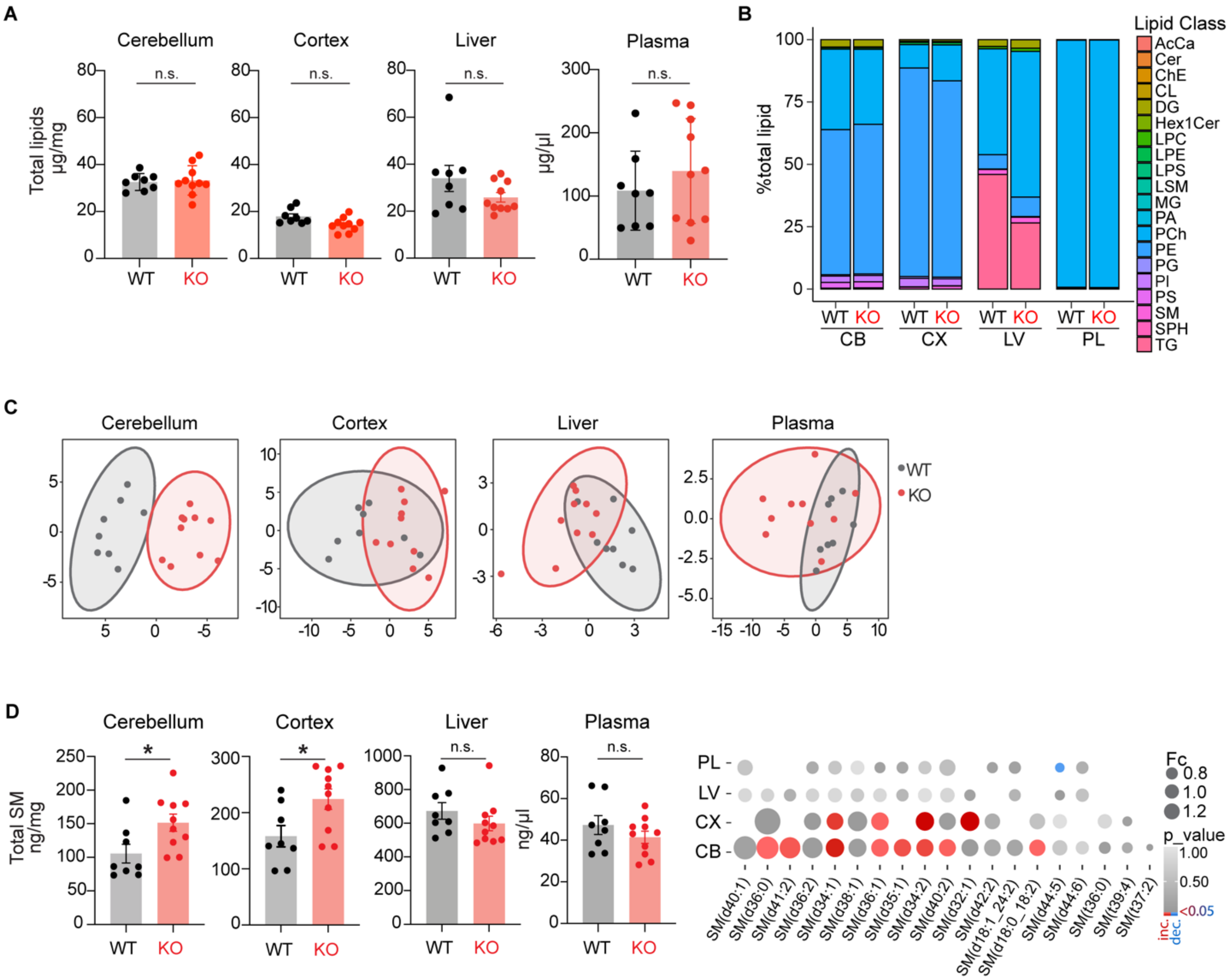


**Supplemental Figure 6. SNX14 deficiency alters lipid metabolite abundance.** (**A**) Bar graphs showing mean ±S.E.M of total lipid concentration in cerebellum, cortex, liver, and plasma of n=8 WT and n=10 KO mice at 2 months of age. Two-tailed student's *t*-test. (**B**) Percentage of each lipid class in indicated tissue of WT and KO mice. (**C**) Principal component analysis of deregulated lipid species separates WT and KO samples in two clusters only in the cerebrum. (**D**) Bar graphs showing mean ±S.E.M of the total SM species concentrations per tissue in n=8WT and n=10KO mice. Two-tailed student's *t*-test. SMs are significantly increased in *Snx14* KO CB and CX. Dot plot depicting FC (proportional to dot size) and p-value (in grey intensity scale) of SM species detected in cerebellar samples for all analyzed tissues. Red dots represent significantly increased lipids and blue dots significantly decreased. n.s. = non-significant, *$P$ <0.05.

Key: AcCa, acylcarnitine; Cer, ceramide; ChE, cholesterol ester; CL, Cardiolipin; DG, diradylglycerolipid; Hex1Cer, hexosyl-1-ceramides; LPC, lysophosphatidylcholine; LPE, lysophosphatidylethanolamine; LPS, Lipopolysaccharide; LSM, lysosphingomyelin; MG, Monoradylglycerolipid; PA, phosphatidic acid; PCh, phosphatidylcholine; PE, phosphatidylethanolamine; PG, phosphatidylglycerol; PI, phosphatidylinositol; PS, phosphatidylserine; SM, sphingomyelin; SPH, sphingosine; TG, triacylglycerolipids.

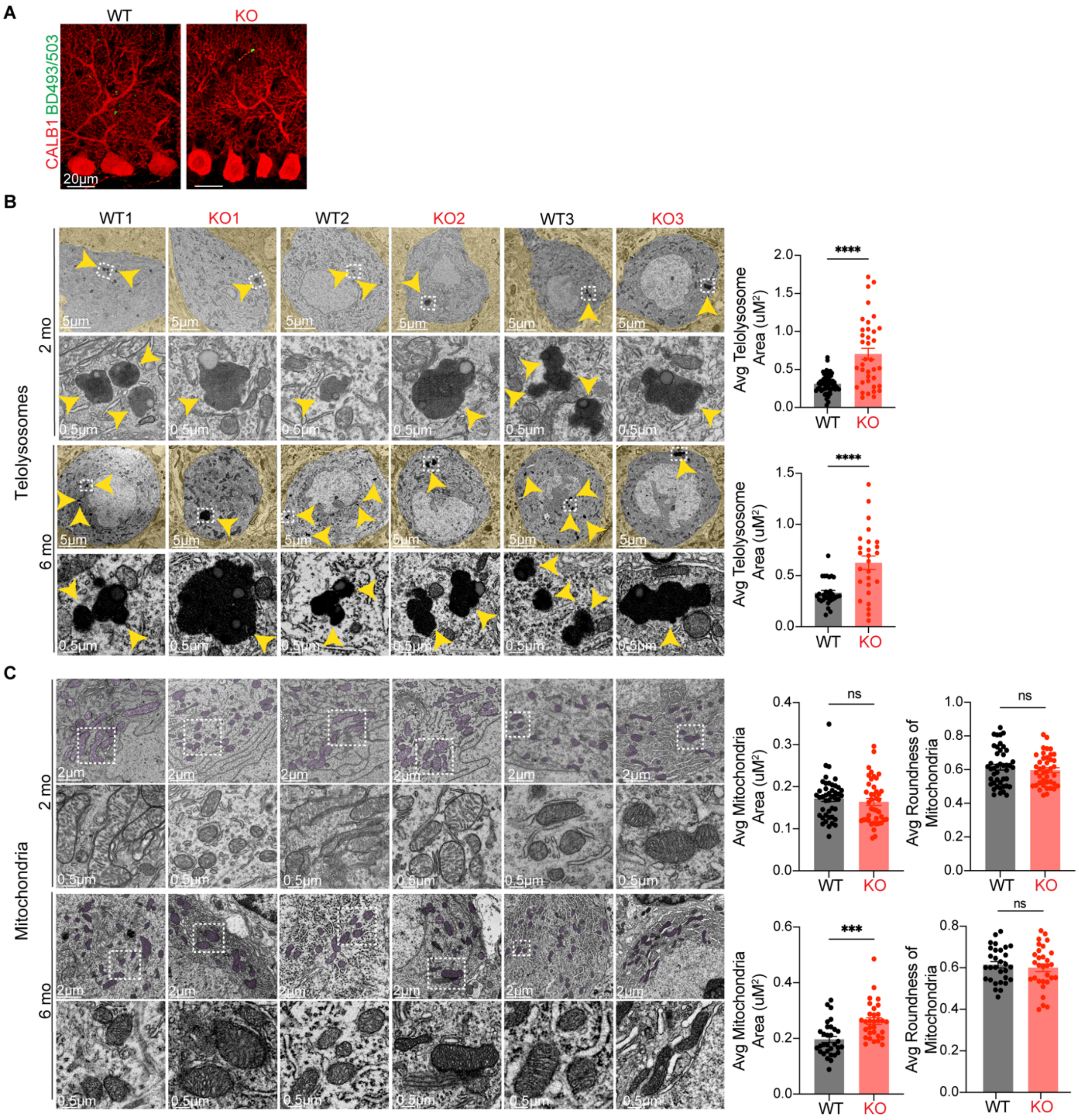


**Supplemental Figure 7. SNX14 KO Purkinje Cells show enlarged telolysosomes and intact mitochondria morphology.** (**A**) Representative image of BD493/503 lipid droplet staining (green) in CALB1 positive PCs (red) of WT and KO cerebellar sections. (**B**) Representative TEM images of telolysosomes in PCs at 2 and 6 months of age. Bar graphs show mean ±S.E.M of the average telolysosome area in N=29 WT and N=26 KO telolysosomes counted from 10 PCs from 3 biological replicates per genotype. Two-tailed student's *t*-test. (**C**) Representative TEM images of mitochondria in PCs at 2 and 6 months of age. Bar graphs show mean ±S.E.M of the average area and roundness of mitochondria N=30 WT and N=30 KO from 10 PCs from 3 biological replicates per genotype. Two-tailed student's *t*-test. n.s. = non-significant, ***$P < 0.001$, ****$P < 0.0001$.
