# Supplementary material for: Altered lipid homeostasis is associated with cerebellar neurodegeneration in SNX14 deficiency
Source: JCI Insight. 2024 Apr 16;9(10):e168594. doi: 10.1172/jci.insight.168594 (PMC11141923; doi:10.1172/jci.insight.168594)

Full unedited gel images for Figure 1A

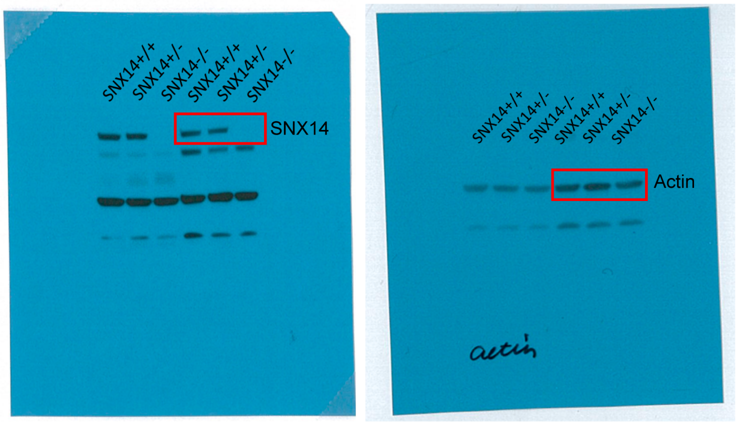

Full unedited gel images for Supplemental Figure 2A

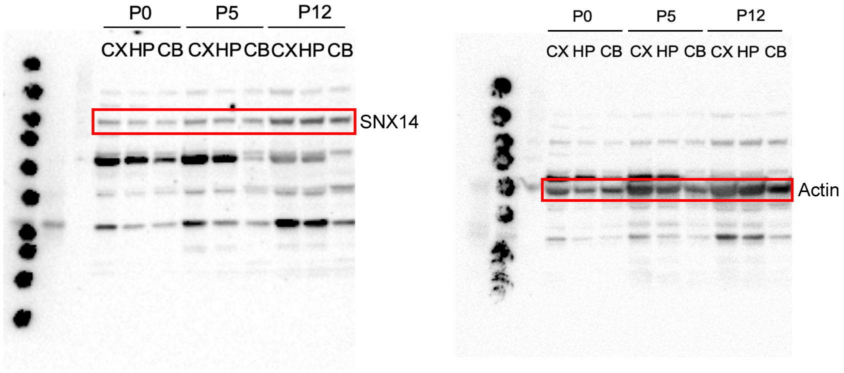

Full unedited gel images for Supplemental Figure 4E

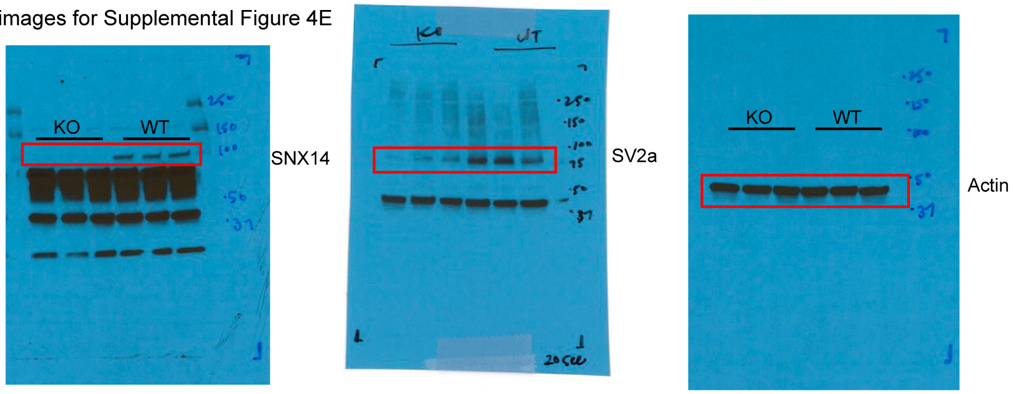

Supplement: Unedited blot and gel images [file jciinsight-9-168594-s191.pdf]
